# Nanoscopic X-ray fluorescence imaging and quantification of intracellular key-elements in cryofrozen Friedreich’s ataxia fibroblasts

**DOI:** 10.1371/journal.pone.0190495

**Published:** 2018-01-17

**Authors:** Björn De Samber, Eline Meul, Brecht Laforce, Boel De Paepe, Joél Smet, Michiel De Bruyne, Riet De Rycke, Sylvain Bohic, Peter Cloetens, Rudy Van Coster, Peter Vandenabeele, Tom Vanden Berghe

**Affiliations:** 1 Department of Analytical Chemistry, Ghent University, Ghent, Belgium; 2 VIB Center for Inflammation Research; Ghent, Belgium; 3 Department of Biomedical Molecular Biology; Ghent University, Ghent, Belgium; 4 Department of Pediatrics, Division of Pediatric Neurology and Metabolism, Ghent University Hospital, Ghent, Belgium; 5 INSERM, Grenoble, France; 6 European Synchrotron Radiation Facility, Grenoble, France; National Institute for Medical Research, Medical Research Council, London, UNITED KINGDOM

## Abstract

Synchrotron radiation based nanoscopic X-ray fluorescence (SR nano-XRF) analysis can visualize trace level elemental distribution in a fully quantitative manner within single cells. However, in-air XRF analysis requires chemical fixation modifying the cell’s chemical composition. Here, we describe first nanoscopic XRF analysis upon cryogenically frozen (-150°C) fibroblasts at the ID16A-NI ‘Nano-imaging’ end-station located at the European Synchrotron Radiation Facility (ESRF) in Grenoble (France). Fibroblast cells were obtained from skin biopsies from control and Friedreich’s ataxia (FRDA) patients. FRDA is an autosomal recessive disorder with dysregulation of iron metabolism as a key feature. By means of the X-ray Fundamental Parameter (FP) method, including absorption correction of the ice layer deposited onto the fibroblasts, background-corrected mass fraction elemental maps of P, S, Cl, K, Ca, Fe and Zn of entire cryofrozen human fibroblasts were obtained. Despite the presence of diffracting microcrystals in the vitreous ice matrix and minor sample radiation damage effects, clusters of iron-rich hot-spots with similar mass fractions were found in the cytoplasm of both control and FRDA fibroblasts. Interestingly, no significant difference in the mean iron concentration was found in the cytoplasm of FRDA fibroblasts, but a significant decrease in zinc concentration. This finding might underscore metal dysregulation, beyond iron, in cells derived from FRDA patients. In conclusion, although currently having slightly increased limits of detection (LODs) compared to non-cryogenic mode, SR based nanoscopic XRF under cryogenic sample conditions largely obliterates the debate on chemical sample preservation and provides a unique tool for trace level elemental imaging in single cells close to their native state with a superior spatial resolution of 20 *nm*.

## Introduction

FRDA is an autosomal recessive disorder with a carrier frequency of 1/100 caused in most cases by a trinucleotide repeat expansion in the *FXN* gene [1]. The latter codes for frataxin, a small mitochondrial iron chaperone involved in iron-sulfur biogenesis, heme biosynthesis and iron storage [2, 3]. The structure and function of frataxin has been extensively studied, including the use of X-ray absorption spectroscopy (XAS) techniques such as near edge structure (XANES) and extended X-ray absorption fine structure (EXAFS) [4–6]. Clinically, the disease is characterized by progressive ataxia, dysarthria, sensory neuropathy, hypertrophic cardiomyopathy and diabetes mellitus. Neuropathologically, the most affected areas are the spinal cord (dorsal nuclei in Clarke columns, dorsal columns, and dorsal spinocerebellar and corticospinal tracts), dorsal root ganglia, dentate nucleus and peripheral nerves [1]. Lamarche et al. were the first to report the presence of granular iron deposits in cardiomyocytes of FRDA patients [7]. Pathophysiologic analysis of tissue samples from FRDA-patients revealed that dysregulation of iron metabolism is a key feature of the disease [8]. For FRDA, the degenerative cell type is mostly expressed in neurons and liver cells, containing relatively high iron concentrations. FRDA has also been suggested to cause redistribution of Fe, Cu and Zn in the dorsal root ganglia [9]. Iron is indispensable in mammalian metabolism and understanding its metabolism gains importance, as there is growing evidence that abnormalities in iron metabolism are involved in the pathogenesis of degenerative diseases [10, 11]. To gain more insight into (neuro) degenerative diseases, iron-catalyzed cell death is an emerging field of study [12, 13]. The redox-active iron pool was found capable of directly catalyzing lipid peroxidation, which results in loss of membrane integrity, ultimately leading to cell death or necrosis [14]. This type of cell death has been called oxytosis, and has recently been analyzed in higher molecular detail and coined as ferroptosis [12, 13]. Although FRDA has been referred to as a prototypic iron-storage disease [15–17], this is still a matter of debate and pathophysiological relevance of the mitochondrial iron loading and the underlying mechanisms are still unknown. Recent results indicate that a modification of iron distribution is a secondary process following Fe-S deficiency that is rather essential to preserve mitochondrial function [15, 18]. Also, it was shown that iron forms aggregates and becomes unavailable for biological processes such as heme biosynthesis [19], which may explain the subsequent increase in iron import and further contribution to mitochondrial iron accumulation. Recently, it is proposed that an excess in cytosolic rather than mitochondrial iron, often referred to as the ‘labile iron pool’ (LIP), is the causative detrimental factor leading to cell death [14, 20]. Other metals such as copper and zinc were also found to be dysregulated in FRDA cells, which has fueled the concept of a more general metal dysmetabolism [9, 21, 22]. The investigation of the spatial distribution of iron and other metals within the subcellular compartments of control and FRDA fibroblasts is therefore of high scientific interest as it may shed light upon the role of metal dysbiosis in FRDA and neurodegenerative diseases in general.

Synchrotron radiation based nanoscopic X-ray fluorescence (SR nano-XRF) is ideally suited to obtain more information on subcellular trace level metal distributions as it provides spatially resolved, (ultra) trace level sensitivity coupled with superior nanoscopic resolution down to 10 *nm* [23]. Due to the highly penetrating character of high-energy X-ray photons, single cells can be examined across their entire depth in a non-destructive manner. The technique is less susceptible to contaminations as minimal sample preparation is required; a counterargument frequently conducted however is that chemical fixation required for in-air XRF analysis of cells modifies their chemical composition. Although nanoscopic computed tomography (nano-CT) of single cells under cryogenic conditions is being routinely performed at low X-ray energies below 1 *keV* [24], routine trace level nanoscopic XRF imaging under cryogenic conditions using hard X-rays was up till now not provided at any synchrotron facility worldwide. In this research paper, we performed the first SR based nanoscopic XRF analysis of single human fibroblasts of control and Friedreich’s ataxia (FRDA) patients under cryogenic temperature using hard X-rays, largely obliterating the debate on the chemical preservation of single cells using chemical fixation procedures. Experiments were performed on the newly installed ID16A-NI ‘Nano-imaging’ setup at the European Synchrotron Radiation Facility (ESRF), providing a hard (17 *keV*) and high flux (2x10^11^ *photons/s*) nanoscopic X-ray beam of 20 *nm*^*2*^.

## Results and discussion

### Prussian blue staining on control and FRDA fibroblasts

**Fig 1:**
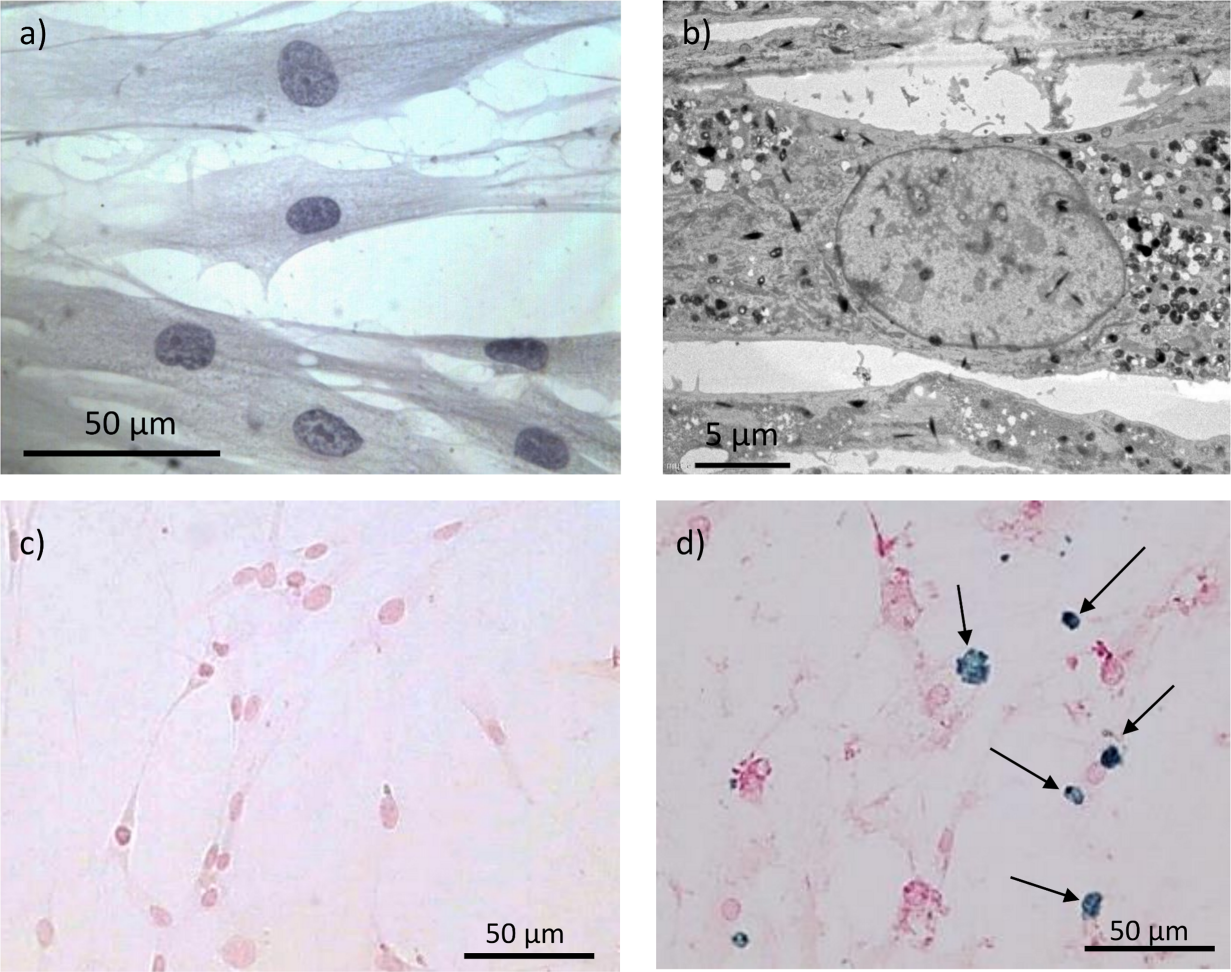
Prussian blue staining and TEM on FRDA fibroblasts. a) Light microscopic image of healthy human fibroblasts; b) TEM image of human fibroblast; c) negative Prussian blue staining of human control fibroblasts; d) positive Prussian blue staining of fibroblasts from Friedreich’s ataxia (FRDA) patients. Iron-rich regions are clearly present in the FRDA fibroblast cells as bright blue regions (indicated with arrows), while fibroblast nuclei and cytoplasm have a red and pink color, respectively.

Fibroblasts were taken from FRDA patients at the University Hospital Ghent (UZ Gent, Department of Pediatrics and medical genetics) using skin biopsy. Since skin biopsies from healthy human volunteers were not available as control due to ethical reasons, fibroblasts from skin biopsies from so called ‘disease-control’ patients were used, i.e. fibroblasts originating from patients tested negative for FRDA and without metabolic disease. In what follows, fibroblast from the ‘disease-control’ group will for simplicity be referred to as ‘control’ fibroblasts. Both control and FRDA fibroblasts were stained with Perls’ Prussian blue staining, more information on the Prussian blue staining method is provided in the Materials and Methods section. Fig 1 shows a light microscopic (a) and TEM (b) image of control human fibroblasts, while (c) and (d) depict Prussian blue stained fibroblasts from control and FRDA patients, respectively. Iron-rich regions are clearly present in the FRDA fibroblast cells as bright blue regions (indicated with arrows) while fibroblast nuclei and cytoplasm have a red and pink color, respectively. The number and size of the iron clumps was strongly dependent on passage number and confluency, and occurred only in a minority of the fibroblasts. The observed presence of iron containing regions in human fibroblasts (mimicking the neuronal phenotype) possibly reconfirms the previously discussed importance of disturbed iron regulation in FRDA [8, 9].

Although straightforward to use, Prussian blue staining for iron staining in fibroblast cells involves several washing and fixation steps, which might cause intracellular elemental redistribution of iron. Since visible light microscopy is used to detect the Prussian blue complex, the technique is limited to the micrometer scale, which complicates subcellular analysis of iron. Although very little is described in the literature on this matter, Prussian blue staining must also have its limitations in terms of sensitivity. In this respect, synchrotron radiation based nanoscopic X-ray fluorescence (SR nano-XRF) is a valuable alternative as it can provide the subcellular trace level iron distribution with superior nanoscopic resolution. Moreover, SR XRF is a multi-elemental technique, probing a large range of elements (e.g. P, S, Cl, K, Ca, Mn, Ni, Cu, Zn) simultaneously. Also, XRF probes elements in a direct manner, which is not the case for Prussian blue staining or fluorescent markers.

**Fig 2:**
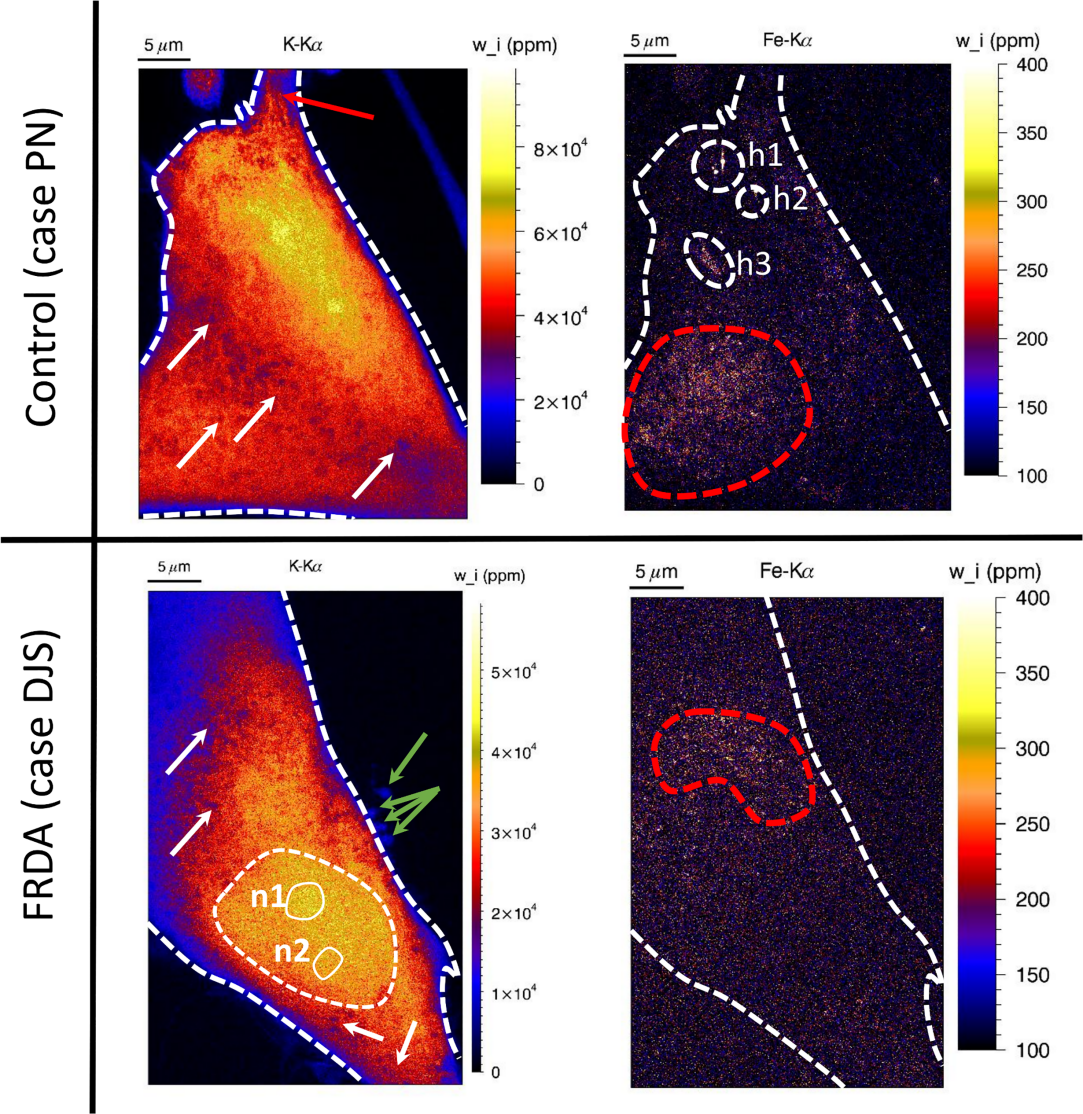
Potassium and iron elemental distribution within control fibroblast case ‘PN’ (upper row) and FRDA fibroblast case ‘DJS’ (lower row). Scale bar indicates the background-corrected mass fraction in ppm, calculated from a mean cell thickness of 10 μm. Pixel size in the images is 55 nm; acquisition time for each pixel is 50 ms. Elemental maps were measured in ‘High dose’ mode and based on detector no. 5 (XIA05) only. All element maps were normalized to dead time, ring current and quantified using the Fundamental Parameter method, taking into account the ice layer thickness determined using the K-Kα/Kβ ratio. Red striped circles indicate areas with iron hot-spots, circles h1-h3 indicate exogenous iron hot-spots, white arrows spherical structures and red arrows fibre-like structures in the fibroblast cells.

**Fig 3:**
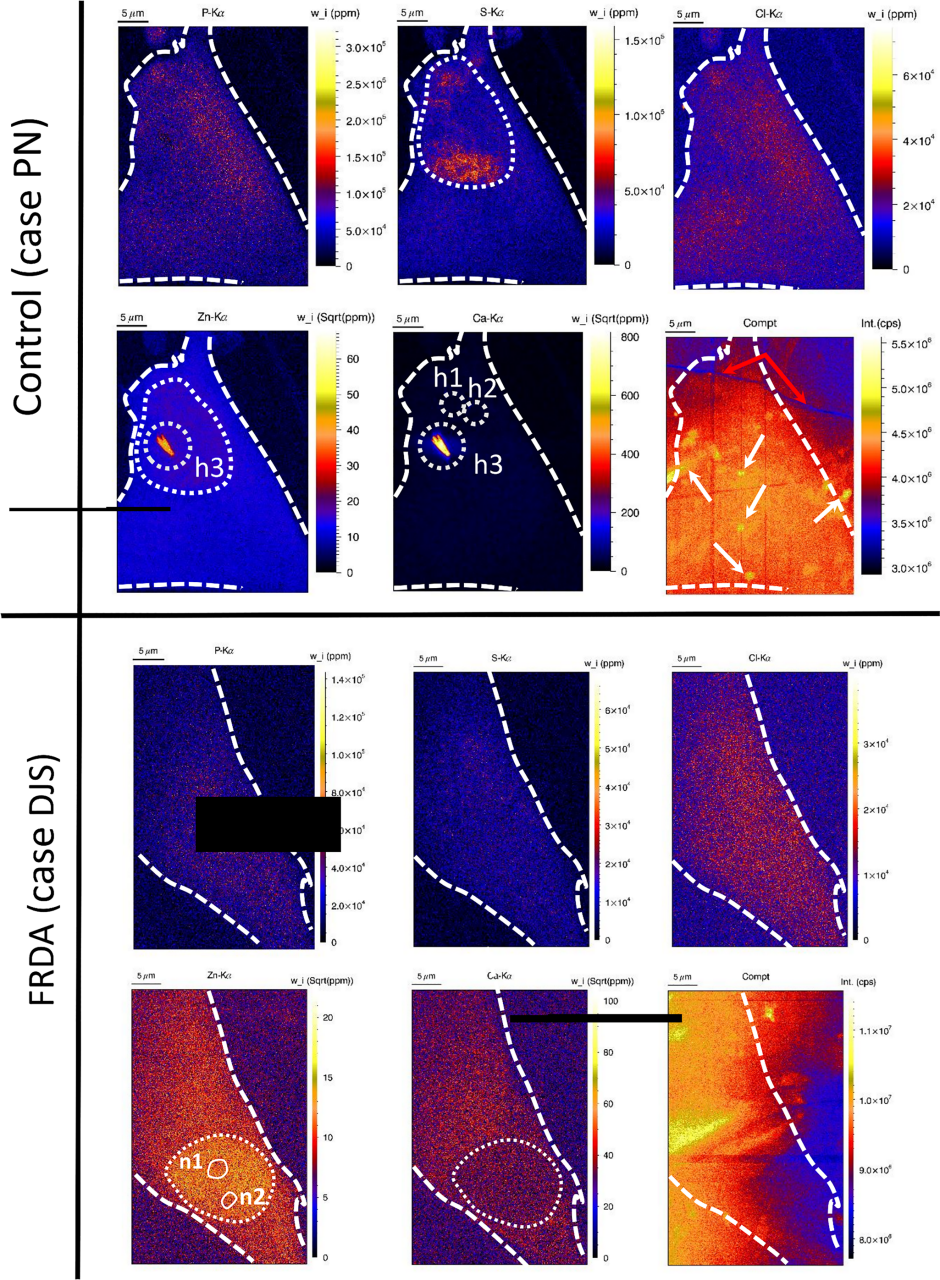
Elemental distribution of P, S, Cl, Ca, Zn and Compton scatter within control fibroblast case ‘PN’ (upper row) and FRDA fibroblast case ‘DJS’ (lower row). Experimental conditions: see legend of Fig 2. White dashed circle indicates the nucleus border. n1-2 indicate the presence of nucleoli.

**Fig 4:**
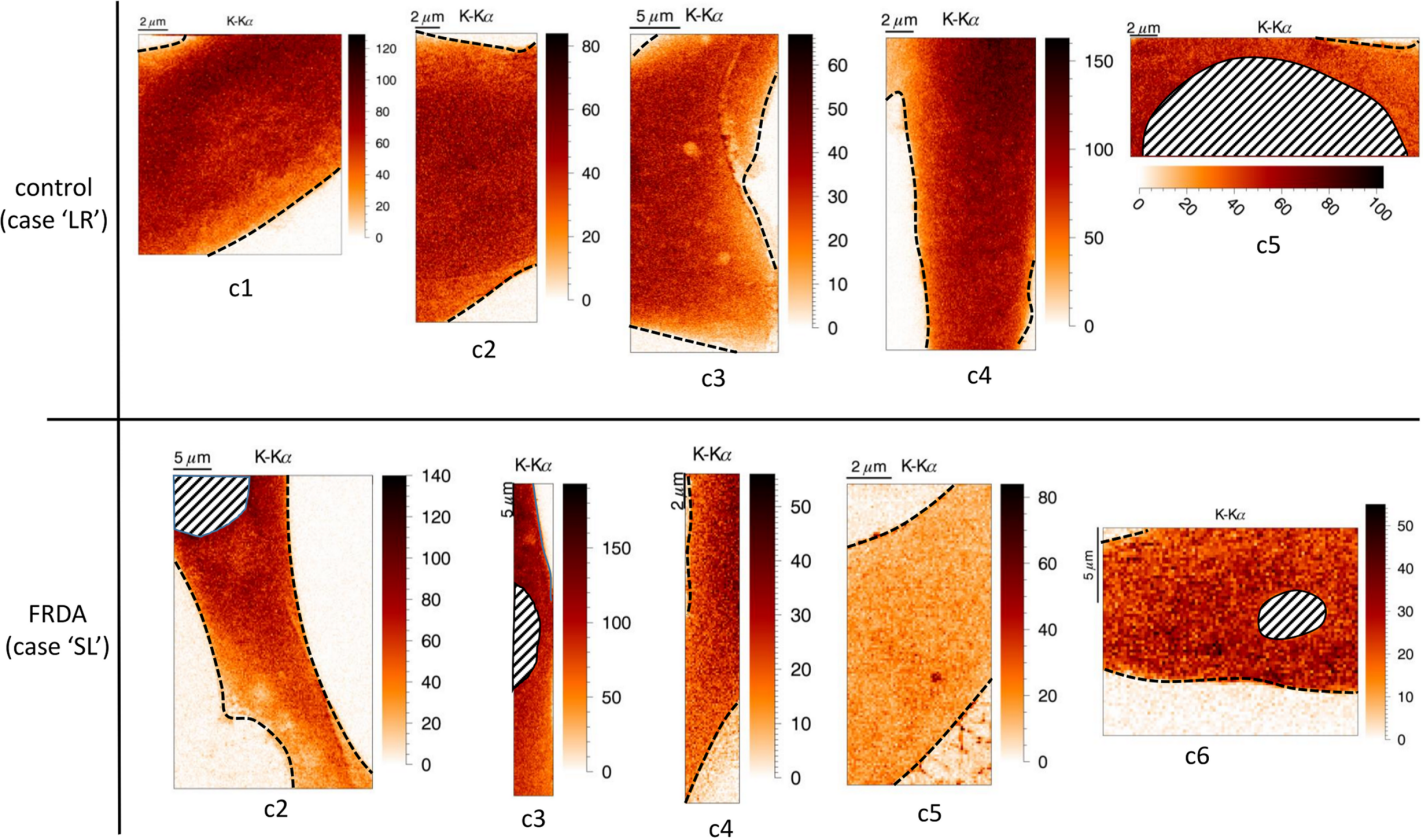
Potassium elemental distribution of (parts of) single fibroblast cells obtained from control patient ‘LR’ and Friedreich’s ataxia patient ‘SL’. For each case, 5 different fibroblasts were scanned. Boundaries of the fibroblasts are indicated, as well as their nuclei (if present). Scans were obtained in ‘Low Dose’ mode, with a step size of 100 nm and a dwell time of 50 ms. Maps are normalized to incoming intensity, 1s measuring time and are dead time corrected.

Although generally a large number of cells need to be measured for biological studies, 2D nanoscopic X-ray imaging under cryogenic conditions is now already capable of mapping tens of cryogenically frozen cells in just a few days of measurement time. Given the large size of fibroblast cells, high resolution nano-XRF scanning of a single fibroblast typically requires 12 *h*. Given the limited amount of experimental measuring time (so-called beamtime) that is assigned at such high-profile instruments (typically 3–5 days), initially only one control (PN) and one FRDA fibroblast (DJS) were scanned at high resolution (55 nm), which is discussed in section ‘Quantification of a single control and FRDA fibroblast’ (Figs 2 and 3). Ideally, fibroblasts from at least five control and five FRDA patients should be measured in biological studies. However, measuring such high number of different patient cells is not feasible: measuring fibroblasts originating from 10 different individuals would currently require 10 cryogenic sample changes during the experiment (requiring 3 hours each) which would result in inefficient use of the precious beamtime allocated. However, a larger number of XRF maps at lower resolution (200 *nm*) of different fibroblasts from the same patient sample can be acquired. In this manner, we could collect 5 XRF maps of another control patient ‘LR’ and 5 XRF maps of another FRDA patient ‘SL’, at a rate of 2–3 hours/fibroblast. These maps were used for investigating the mean elemental concentration of the cytoplasm accompanied by statistical analysis in section ‘Quantitative comparison of cytoplasm mean mass fractions of 5 control and 5 FRDA patient fibroblasts’ (Fig 4).

### Quantification of a single control and FRDA fibroblast

Fig 2 compares the background corrected, mass fraction elemental maps of iron and potassium in parts-per-million (or *ppm*) of a randomly selected fibroblast from control patient ‘PN’ (upper row) with those of a fibroblast from FRDA patient ‘DJS’ (lower row). Scanning conditions were identical for both fibroblasts, i.e. 55 *nm* step size and 50 *ms* dwell time. For obtaining highest elemental sensitivity, fibroblasts were measured in ‘high dose’ (HD) mode, i.e. with highest X-ray flux achievable of 2.10^11^ *photons/s*. Mass fraction (quantity indicated with ‘w_i’ in the graph) was calculated assuming a constant 10 *μm* thin cell layer. A single SDD detector was used for quantification (for more information see Materials and Methods section, ‘ID16A-NI detector optimization’). After estimating the ice layer thickness for both cases, quantitative elemental maps were determined using the Fundamental Parameter equation (see Materials and Methods section, ‘XRF quantification’: ‘Determination of ice layer thickness’ and ‘Determination elemental mass fractions within single, cryofrozen cells’). For the sake of clarity, the outer boundaries of both fibroblasts are denoted by a white striped line. Iron containing hot-spots (indicated with h1-h2) which were found in the control fibroblast (indicated with h1/h2) are likely non-biogenic due to their high countrate. To better visualize the ‘true’ biogenic iron with lower mass fraction, maximum of the color bar of the iron elemental map was set to 400 *ppm*. Also, to remove a still luminous background (despite the effected background correction), the minimum of the iron mass fraction bar for the control and FRDA case was set at 100 *ppm*. Using this optimized scaling, we observe areas surrounding the fibroblast nucleus rich in iron hot-spots, indicated in Fig 2 by the areas encircled by a red striped line. Both the control and FRDA fibroblast contained such iron hot-spots, with a similar average mass fraction of approx. 200 *ppm* (equivalent to 3.5 *mM*). It is currently unknown whether the iron containing hot-spots are residing within the cytosol or within organelles such as mitochondria or lysosomes. Iron remains however undetected in areas in the elemental map where its local concentration is below the limit of detection of (7.6 *ppm* or 140 *μM*). Provided its higher concentration, potassium clearly outlines the detailed morphology of both fibroblast cells. Although assumed to be homogeneously distributed, the cytosol of both fibroblast cases is littered with sub-micrometer sized spherical regions poor in potassium and rich in chlorine (indicated by white arrows in Fig 2). For the control fibroblast only, we observe the presence of fiber-like structures (red arrows). For the FRDA case, micrometer-sized spherical structures are present close to the cell membrane (green arrows), which are somewhat too large to be exosomes (max. size up to 100 *nm*). The absence of iron enrichments in FRDA fibroblast cells compared to the control fibroblasts as observed with Prussian blue staining discussed in previous section may have different causes. First, after skin biopsy fibroblasts were cultured *in vitro* for several cycles inferring an unnatural environment in which the cells could already behave different compared to *in vivo* conditions. Another reason may be that due to the limited degree of sampling, only fibroblasts were measured where no iron accumulation was present. In this regard, preselection of the candidate fibroblasts containing a high degree of iron using fluorescence-activated cell sorting (FACS) would be a meritorious effort, given that fluorescent labels with sufficient iron-sensitivity are available.

Fig 3 shows the corresponding mass fraction elemental maps of P, S, Cl, Zn, Ca (*in ppm*) and Compton scatter (in *counts per second* or *cps*) of the same control fibroblast case ‘PN’ (upper row) and FRDA fibroblast case ‘DJS’ (lower row). For better distinction between the different concentration levels, square rooted mass fraction element maps of zinc and calcium are shown. Zinc is homogeneously present in the cytoplasm, but present in slightly higher concentration in the nucleus. In the control fibroblast, a zinc hot-spot is present (indicated with h3 in Fig 3) with coinciding presence of calcium and iron. Experience has shown that the simultaneous presence of these elements is often a contamination (e.g. salt crystal, dust particle) and therefore the Ca/Fe/Zn-containing hot-spot is most likely exogenous. For the FRDA fibroblast, a lower zinc mass fraction is found in two spheres within the nucleus, which are likely nucleoli (indicated with n1-2 in Fig 3). Note that for the FRDA case, the cytosolic zinc mass fraction is significantly lower compared to the control case. Decrease of the zinc mass fraction in the cytosol of FRDA fibroblasts may be explained by impaired zinc homeostasis associated to FRDA [21]. Interestingly, sulphur-rich areas are present in the control fibroblast only, indicated with dashed circle in Fig 3. Since Fe-S clusters were found to play an important biological function, SR-XRF shows its potential by probing several elements simultaneously [14]. Quantification and interpretation of sulphur distribution is however risky to interpret due to the heavy absorption of the low energy S-K_α_ fluorescent photons in the ice layer covering the cell (see also Materials and Methods section, ‘Quantification’, ‘Determination of ice layer thickness’).

As the fibroblasts were scanned at high resolution, mean elemental concentration values could be determined within entire fibroblast and well-distinguishable regions of nucleus and cytoplasm. The quantification provided additional information on elements present in fibroblasts, but below detection level in scanning mode (e.g. for manganese and copper) and therefore not observable in the element maps. Table 1 shows a quantitative comparison of a single fibroblast from control patient ‘PN’ and FRDA patient ‘DJS’ with ice layer thickness correction using the ‘K-K_α_/K_β_’ method (see earlier). All concentration values were background corrected; molar concentrations were calculated assuming an ice matrix. As previously observed in Fig 1, mean iron concentration in both entire fibroblasts remains constant around 50 *ppm* (approx. 1 *mM*), no local iron increase is observed in the nucleus nor in the cytosol either. Interestingly, we observe a strong zinc concentration decrease for the FRDA fibroblasts of almost a factor of two, both in the entire FRDA fibroblast as well as in the nucleus and cytoplasm. Mean zinc concentrations in entire cell, nucleus and cytoplasm decrease to 52, 12 and 30 *ppm*, respectively (corresponding with 800, 180 and 410 *μM*). For manganese, we observe a slight overall concentration increase for the FRDA fibroblast: from 16 to 24 *ppm* (corresponding with 290 and 440 *μM*, respectively). For the elements Mn, Fe, Ni and Cu, no quantitative values for the nucleus are provided as background subtraction resulted in negative concentration values. Since here, concentrations within a single control and FRDA fibroblast were determined, the interpretation of concentration differences between control and FRDA fibroblasts is only conducted in the following section, where the quantification procedure was applied for 5 control and 5 FRDA cells, providing additional statistical information. Note that the obtained intracellular concentrations are generally somewhat higher than those obtained for XRF quantification in human neutrophils obtained in earlier work [25]. However, for the latter case no cryogenic sample environment was available and therefore cells were high pressure frozen and cryosubstituted in acetone. During this process, unbound metals can migrate and, in this way, cause lower concentrations. Other causes for differences in concentration include: estimation of the ice layer thickness above the fibroblasts, calibration using a standard reference material involving areal mass estimation and absorption/inhomogeneities in the standard reference material.

**Table 1:** Background-corrected mean mass fractions of the elements P, S, Cl, K, Ca, Mn, Fe, Ni, Cu and Zn within 1) entire cell body 2) nucleus and 3) cytoplasm of a single fibroblast from control patient ‘PN’ and Friedreich’s ataxia patient ‘DJS’.

		Mean concentration (background corrected) (*w%*, *ppm* or *μM*)							
		control (case PN)				Friedreich's ataxia (case DJS)			
		Entire cell	Nucleus	Cytoplasm	Backgr.	Entire cell	Nucleus	Cytoplasm	Backgr.
Mass fraction, *%*	**P**	3.66	0.92	2.31	0.24	1.19	0.20	0.74	0.00
	**S**	1.54	0.50	0.89	0.08	0.47	0.07	0.32	0.08
	**Cl**	1.13	0.13	0.71	0.23	0.61	-	0.38	0.25
	**K**	**2.82**	0.84	1.80	0.04	**1.47**	0.49	0.96	0.02
Mass fraction, *ppm* (*μM*)	**Ca**	**697 (17400)**	160 (4000)	**431 (10800)**	85	**525 (13100)**	-	**361 (9000)**	179 (4460)
	**Mn**	**16.1 (293)**	-	**9.57 (174)**	6.3	**24.2 (440)**	-	**14.6 (265)**	15.3 (278)
	**Fe**	**52.8 (946)**	1.68 (30)	**33.3 (595)**	15	**51.9 (928)**	-	**32.7 (585)**	29.1 (522)
	**Ni**	0.33 (5.7)	-	0.19 (3.3)	0.2	0.63 (11)	-	0.40 (6.8)	0.39 (6.7)
	**Cu**	**14.0 (220)**	-	**8.23 (129)**	6.0	**20.5 (323)**	-	**12.3 (193)**	13.2 (208)
	**Zn**	**98.9 (1513)**	32.3 (495)	**57.7 (882)**	4.7	**51.8 (792)**	11.6 (177)	**29.9 (457)**	10.1 (155)
**Ice layer thickness**	**K-Kα/Kβ**	7.6 μm				4.0 μm			
	I_0_/I_t_	14 μm				50 μm			

XRF spectra from detector no. 5 only were used only for quantification. Mass fractions are expressed in *w%* for P, S, Cl, K and in *ppm* for Ca, Mn, Fe, Ni, Cu and Zn; values in parentheses are in *μM*. Background concentration was determined as the mean concentration value of an area defined well outside the cell. Absorption correction for the ice layer covering the fibroblasts was carried out using the K-K_α/β_-method. For the elements Mn, Fe, Ni and Cu concentration, no quantitative values for the nucleus are provided as background subtraction results in negative value. Mass fractions (molarities) of interest are indicated in bold and discussed in the manuscript text.

### Quantitative comparison of cytoplasm mean mass fractions of 5 control and 5 FRDA patient fibroblasts

Since 5 fibroblasts from control patient case ‘LR’ and 5 fibroblasts from FRDA patient case ‘SL’ were scanned with relatively large step sizes of 200 *nm* and 100 *ms* dwell time per point, quantification and statistical analysis was performed on these 2 cases. To reduce radiation damage during these initial scans, cells were measured in ‘low dose’ (LD) mode, i.e. a lower beam flux of *3*.*35 10*^*10*^ *photons/s* obtained by placing a 4.6 μm thin Au foil in the beam path. Fig 4 shows the normalized net K-K_α_ intensity maps of all 10 fibroblasts. Boundaries of the fibroblast cells and presence of nuclei are indicated. Since in most of the fibroblast scans, only the cytoplasm (comprising the cytosol and organelles) was present, only this part of the fibroblast could be quantified. This however posed no significant problem as the main aim of this study was quantification of cytosolic or subcellular iron and other key metals like zinc and copper. Quantification was applied as described in Materials and Methods, ‘Determination elemental mass fractions within single, cryofrozen cells’. Table 2 shows the background-corrected mean concentrations within the cytoplasm of 5 single fibroblasts from control patient ‘LR’ and 5 fibroblasts from FRDA patient ‘SL’. Values are expressed in *w%* for the elements P, S, Cl and K; in *ppm* for Ca, Mn, Fe, Ni, Cu and Zn; values between parentheses are in *μM*. Relative standard deviation (RSD) for each element mass fraction is provided in %. The RSD of the mass fractions is above 50% in most of the cases, indicating a rather large spread in determined concentration values for the cytoplasm. Also the RSD of the ice layer thickness covering the FRDA case fibroblasts determined by the K-K_α_/K_β_ ratio is larger compared to the control case: approx. 70% vs 4%, showing large sample-dependent differences in spread of ice layer thickness. Using the statistical software package SPSS 23 (IBM), a probability-probability (P-P) plot showed however that cytoplasm mass fractions from both control and FRDA patient fibroblasts are normally distributed for (nearly) all elements across the total number of cells scanned. This could be considered as an indicator that a representative number of fibroblasts grown upon the (same) Si_3_N_4_ wafer was analyzed. Given that the mass fractions are distributed normally, an (independent samples) Student’s T-test was made to verify whether mean mass fraction values of control and FRDA fibroblasts are significantly different or not. Note that the share of the background with respect to the background corrected mass fraction is above 50% for a majority of elements; such increased background level increases the limit of detection (LOD) and impairs the detection of significant mass fraction differences below the increased LOD level.

**Table 2:** Background-corrected mean mass fractions of the elements P, S, Cl, K, Ca, Mn, Fe, Ni, Cu and Zn in the cytoplasm of 5 fibroblasts from control case ‘LR’ and FRDA case ‘SL’.

		Control patient 'LR' (n = 5 cells)				Friedreich's ataxia patient 'SL' (n = 5 cells)				Student T-test (Equality of means)	Levene's test (Equality of variances)
		Mean conc. cyt.	RSD	Backgr.	Normality	Mean conc. cyt.	RSD	Backgr.	Normality	2-tailed Sign.	Sign.
		(w%, ppm or *μM*)	(%)	(%)		(w%, ppm or *μM*)	(%)	(%)		90% conf. int.	90% conf. int.
Mass fraction, %	P	3.89	56	0.9	++	6.04	160	5.3	++	0.651	0.085
	S	1.00	57	0.8	+	1.17	124	3.3	++	0.810	0.174
	Cl	1.40	52	**10.4**	++	1.28	107	**73.1**	+	0.875	0.373
	**K**	**1.90**	68	0.3	++	**0.90**	123	3.0	++	**0.227**	0.745
Mass fraction,	**Ca**	**518 (12900)**	37	1.2	+	**296 (7390)**	63	7.6	**+++**	**0.102**	0.958
*ppm* (*μM*)	**Mn**	**12.0 (219)**	48	9.9	+	**9.0 (163)**	52	**59.6**	++	0.386	0.38
	**Fe**	**39.3 (704)**	41	**10.5**	++	**37.3 (669)**	63	**60.1**	++	0.883	0.756
	Ni	0.21 (3.6)	47	**10.4**	++	0.16 (2.8)	52	**63.4**	++	0.467	0.481
	Cu	12.1 (190)	50	**11.9**	++	9.93 (156)	52	**74.1**	++	0.565	0.449
	**Zn**	**39.1 (598)**	63	2.1	+	**21.4 (328)**	37	**8.3**	**+++**	**0.188**	0.018
Ice layer thickness (μm)		**6.26**	4		+	**7.1**	66			0.698	0.052

Mass fractions are expressed in *%* for P, S, Cl, K; in *ppm* for Mn, Fe, Ni, Cu and Zn and values in parentheses in *mM*; relative standard deviation (RSD) is expressed in %. Only XRF spectra from detector no. 5 were used for quantification. Mean ice layer thickness (in *μm*) for each case (‘LR’ and ‘SL’) was determined using the K-K_α_/K_β_ method, its associated RSD value (in %) is indicated at the bottom of the table. ‘Share background’ represents the ratio (in %) of the background mass fraction to the background corrected mass fraction of the cytoplasm for a given element. ‘Normality’ refers to the extent to which a normal distribution is present among the concentration values of the different cells (‘+++’: perfect normality for all values, ‘++’: close to normal distribution, slight asymmetry or 1 outlier, ‘+’: 2 outliers or larger asymmetry). Student T-test verifies the equality of the mean concentration values of control case ‘LR’ and FRDA case ‘SL’ with 90% confidence interval; significance (or *p*) values closer to 0 (indicated in bold) indicate a significant difference at a 90% confidence interval (K, Ca and Zn). Levene’s test indicates the equality of variances of mass fractions for case ‘LR’ and ‘SL’; larger values (indicated in bold) indicate equal variances. Concentration values of elements discussed in the manuscript text are indicated in bold as well.

For potassium and calcium, we observe a significant decrease (at the 90% significance level) in mean cytoplasm concentration when comparing control with FRDA fibroblasts: from 1.90 to 0.90% and from 518 to 296 *ppm* (equivalent to 12.9 *mM* to 7.4 *mM*), respectively. Note however that the uncertainties of the absorption correction for the ice layer thickness of the control and FRDA cytoplasm mass fractions for lower atomic number elements such as P, S, Cl, K and Ca may already result in significant mass fraction differences between both cases. This is not the case for higher atomic number elements such as Mn, Fe, Ni, Cu and Zn, which requiring almost no absorption correction factor for the ice layer (see Materials and Methods, ‘Determination elemental mass fractions within single, cryofrozen cells’ and S7 Fig). Similar to the comparison of a single control and FRDA fibroblast in previous section, we found no significant difference between the iron concentration of the background-corrected mean mass fraction of the cytoplasm of control and FRDA fibroblasts. Mean iron mass fraction in the cytoplasm of FRDA fibroblasts was however slightly lower compared to control fibroblasts: 37.3 *ppm* vs. 39.3 *ppm* (or 669 vs 704 *μM*). Although not significant, we also observed a slight decrease in cupper concentration (156 vs 190 *μM*). It was recently found that copper levels were significantly lower in hearts of FRDA patients, which is considered to be important for the potential benefit of copper supplements in FRDA cardiomyopathy [21]. As for the single control and FRDA fibroblast quantification (see previous section), we reconfirm a significant decrease of mean zinc concentration in the cytoplasm of FRDA fibroblasts: 39.1 versus 21.4 *ppm* (equivalent to 598 vs 328 *μM*). XRF analysis of polyethylene glycol-embedded dorsal root ganglia (DRG) of FRDA patients by Koentzsch et al. revealed abundant presence of zinc also at micromolar level [22]. There, it was hypothesized that inappropriate release of free zinc from the large pool of protein-bound zinc damages neuronal mitochondria since the observed zinc concentration constitutes a large excess over the estimated picomolar requirements of zinc-dependent cellular proteins.

## Materials and methods

### Skin biopsy, cell culture and Prussian blue staining

Fibroblasts were taken from FRDA patients at the University Hospital Ghent (UZ Gent, Department of Pediatrics and medical genetics) using skin biopsy. The ethics committee of the Ghent University Hospital (UZ Gent) approved the research project and the associated collection of skin biopsies. The research was declared to the Commission for the Protection of Privacy (CPP, Belgium). Since skin biopsies from healthy human volunteers were not available as control due to ethical reasons, fibroblasts from skin biopsies from ‘disease-control’ patients were used. Skin biopsies from patients ‘PN’ and ‘LR’ from the disease-control group were tested negative for FRDA. Fibroblast cells were obtained from FRDA patients ‘DJS’ and ‘SL’. Skin biopsies from Friedreich’s ataxia and disease-control patients were collected with prior consent of patients and no money has been exchanged in whatsoever form to collect the samples. After skin biopsy, human fibroblasts were further cultured in Opti-MEM™ medium supplemented with 1% L-glutamine solution, 1% Penicillin Streptomycin solution, and 12.5% Fetal Bovine Serum and kept in an incubator at 37°C and 5% CO_2._

For Prussian blue staining, collected fibroblasts were grown on glass slides and air-dried, fixed in methanol for 10 min and washed with distilled water. Equal volumes of hydrochloric acid (HCl, 20% aqueous solution) and potassium ferrocyanide (K_4_Fe(CN)_6_.3H_2_0, Sigma Cat# P-3289, 10% aqueous solution) were mixed for immediate use. Slides were immersed in this solution for 30 minutes and then washed three times in distilled water. Dilute mineral acid hydrolysis releases ferric (Fe^3+^) ions, which in the presence of ferrocyanide ions [Fe^II^(CN)_6_]^4-^, is precipitated as the bright blue and highly water-insoluble complex, potassium ferric ferrocyanide KFe^III^Fe^II^(CN)_6_ or Prussian blue:

$${\mathrm{FeCl}}_{3}+K_{4}{\mathrm{Fe}}^{\mathrm{II}}{\left(\mathrm{CN}\right)}_{6}\to {\mathrm{KFe}}^{\mathrm{III}}{\mathrm{Fe}}^{\mathrm{II}}{\left(\mathrm{CN}\right)}_{6}↓+3\mathrm{KCl}$$

Afterwards, slides were counterstained with nuclear fast red for 5 min, rinsed twice in distilled water before dehydration.

### Sample preparation for SR nano-XRF

For SR nano-XRF analysis, fibroblast cell cultures were transported to the ESRF and immediately brought to incubator at 37°C with 5% CO_2_ level available at the containment level 2 lab (ESRF biomedical facility, cellular biology) equipped with centrifuge, light microscope, analytical balance, fridge at 4°C and freezer at -20°C. Under previously described culture conditions (considered as ideal), fibroblasts were grown upon Si_3_N_4_ wafers considered as ideal support for XRF analysis (from Silson Ltd., Northampton (UK), 5x5 *mm*^*2*^ size, 1.5 *mm*^*2*^ membrane area, 500 *nm* membrane thickness and 200 *μm* frame thickness). After verification of firm attachment and spreading of the fibroblasts using optical microscopy, wafers were taken to the sample preparation laboratory using an autoclavable ‘bio-carrier’ red box. There, Opti-MEM™ cell culture medium was removed, wafers briefly washed in 0.25 *M* freshly prepared ammonium formate solution to remove salts and trace metals from the medium (3.1538 *g* ammonium formate Optima® LC/MS grade, Fisher Chemical™ dissolved in 200 *mL* milli-Q™ water). As vitreous ice upon the fibroblasts causes X-ray absorption, excess water is removed before plunge freezing via blotting. Wafers were blotted and then plunge frozen in liquefied ethane automatically using a Leica EM GP™. After plunge freezing, a cryogenic workflow is maintained until and during the XRF measurements. Wafers are transferred into the liquid nitrogen bath of a Leica™ EM VCM (Vacuum Cryo Manipulation System) where they are clamped in a pre-cooled gold-coated VCT sample holder. The sample holder can then be loaded into the Leica™ EM VCT500 (Vacuum Cryo Transfer system). Finally, sample shuttle is attached to the ID16A-NI vacuum chamber and the gold-coated copper cube holding the silicon nitride membrane with deposited fibroblasts is transferred onto the sample stage kept at -150*°C*. An external visible light microscope system with long working distance positioned outside the vacuum chamber is used to bring fibroblast cells in the focus of the X-ray nanobeam.

During our analyses, fibroblasts generally remained well-preserved during scanning XRF, nevertheless even under cryogenic conditions we did observe sample radiation effects such as slow freeze-drying of the sample or abrupt ablation of the sample, typically occurring for cells covered by a thinner ice layer of a few micron. Under these circumstances, parts suddenly detach from the fragile, glass-like frozen cell layer when struck by a high intensity X-ray nanobeam. Higher sensitivity of the sample with respect to radiation damage was related to a lower ice layer thickness covering the cell, i.e. a cell covered by an ice layer of only a few micron is less protected than a cell covered by several tens of microns of ice.

### ID16A-NI ‘Nano-Imaging’ beamline

Scanning nano-XRF experiments were performed at the ID16A-NI (Nano-Imaging) beamline (UPBL04) at the European Synchrotron Radiation Facility (ESRF). ID16A-NI is a 185 *m* long beamline for hard X-ray microscopy, providing quantitative 3D characterization at the nanoscale of the morphology and the elemental composition of specimens in their native state [23]. The beamline provides the world’s brightest hard X-ray nanofocus, i.e. 2 x 10^11^ *photons/s* confined within a beam of 27 *nm* horizontally by 21 *nm* vertically full-width at half-maximum (FWHM). Incident energy upon the X-ray focusing optics housed within the high vacuum sample chamber is 17.1 *keV* having a spectral bandwidth *ΔE/E* of 1*%*.; also 33.6 *keV* excitation energy can be provided. Beamline techniques include full field holography, ptychography and X-ray fluorescence. Measurements are always performed vacuum whereas cryogenic analysis is optionally. In the latter case, temperature of the sample holder clamping the sample wafer is continuously monitored and generally remains constant at -150°C. Main fields of application are life science and nanotechnology. A positioning stage provides nanoscopic positioning and fast continuous scanning with maximal speed of 4 *μm/s*. A diode is present behind the sample determining the flux density for normalization purposes. The X-ray nanobeam can be provided in ‘high-dose’(HD) and ‘low-dose’ (LD) mode; in HD-mode, a thin Au foils of 4.6 μ*m* is removed, increasing the photon flux by a factor of 3.1 times to 2.10^11^ *photons/s* with respect to LD-mode.

### ID16A-NI detector optimization

Two pairs of three vertically aligned silicon drift detectors (SDDs, SGX Sensortech) were connected to left vacuum flange (downstream direction) of the sample chamber, orthogonal to the beam direction. All six SDDs are equipped with a beryllium window and have 50 *mm*^*2*^ active area each. Minimal detector distance was set at 40.2 *mm*, limited by the experimental geometry at that time. Due to the vertical alignment of the detectors, not all SDD detectors could be placed in the synchrotron ring plane where Compton scatter is minimal under 90° detector angle. In order to have the sample surface perpendicular to the X-ray beam and still being able to collect X-ray fluorescence emerging from the sample, the angle between sample surface and detector was set to approx. 18°. A shielding plate was attached to the detector to protect it from scattered X-ray photons from the mirror system; no collimator was used.

S1 Fig shows the elemental maps of P, S, Cl, K, Ca, Cr, Mn, Fe, Ni, Cu, Zn, Sr, Au, Rayleigh and Compton scattering (separately for each single SDD detector, numbered from 0 to 5) for FRDA fibroblast, case ‘DJS’, measured in HD. A 50 *ms* dwell time per pixel and 55 *nm* step size was used. Since the total scanned area covers 44.9 x 32.7 *μm*^*2*^, each element map contains 816 x 595 pixels and was derived from 480k individual XRF point spectra. For potassium, we clearly observe the outlined fibroblast for SDD detector no. 3, 4 and 5 only (indicated with green box no. 1 in S1 Fig). The reduced signal intensity for detector no. 0, 1 and 2 is most likely due to their less favorable position. Interestingly, the nickel, copper and gold element maps from SDD detector no. 5 (green box no. 2 in S1 Fig) show a lower intensity and less structure compared to that of the other detectors (red box no. 2 in S1 Fig). The inhomogeneous distribution of nickel, copper and gold maps are not indicating the presence of these metals in the fibroblast, but can be explained by the presence of microcrystals within the vitreous ice matrix. More specifically, when raster-scanned, photons impinging on these crystalline areas are diffracted and excite the gold-coated cupper sample holder and nickel present in the inner parts of sample chamber, generating additional background and secondary fluorescence not originating from the sample itself. Because cryogenically frozen cells are mainly composed of ice, they show a significant increase in scattered X-ray photons compared to previously widely used methods for single cell analysis in non-cryogenic mode such as embedding or freeze-drying. The Rayleigh (inelastic) scattering map bears close resemblance to the gold elemental map, confirming that gold fluorescence is caused by elastic scattering. This phenomenon is unwanted as it causes the virtual presence of elements in the elemental maps. The presence of microcrystals in the samples is surprising as it is generally assumed that during the plunge freezing procedure, all water is immediately converted into amorphous ice. Diffraction occurring in the microcrystals present in the cryogenically frozen cells could be overcome by finding shock-freezing methods preventing the formation of microcrystalline areas. Secondary fluorescence of gold and nickel reaching the detector could be eliminated via the use of collimators and/or multilayer coatings with increasing atomic number. Another measure currently in commissioning phase at beamline ID16A-NI is a dual detector array setup providing mutual shielding, which is expected to improve the limit of detection (LOD) for iron in scanning mode by at least a factor of 10, i.e. well below the *ppm* level. The Compton scattering also reveals the location of microcrystals, but is also indicating the presence of ice cracks as it is representative for the electron density of the cell covered with ice. The zinc elemental map of SDD detector no. 5 correlates better with the morphology of the fibroblast itself (indicated with green box no. 3 in S1 Fig), which might be related to the fact that detector no. 5 is positioned in such a manner that it collects less secondary fluorescence. Distinct hot-spots of iron—element of main interest—in the fibroblasts are more apparent in the element map of detector no. 5. Also elemental distributions of phosphorous, sulphur and chlorine are less noisy in detector no. 5 (green box no. 4).

S2 Fig shows the elemental maps of iron and potassium for SDD detector no. 3, 4 and 5 in higher detail for the same FRDA fibroblast shown in Fig 2. For clarity, the cell boundaries as deduced from the potassium distribution are marked upon the iron elemental maps. We observe iron hot-spots in the elemental map obtained from SDD detector no. 5 only (encircled in red in S2 Fig), which would–given the count rate—disappear in the background signal when adding the other detector intensities for iron. Also for chlorine, calcium and zinc, a clear increase in signal-to-noise ratio for was observed for detector no. 5. As the investigation of iron among other metals was an important aim in this study, only XRF data from detector no. 5 (or XIA05) was used for imaging and quantitative analysis.

Additionally, we investigated the dead time map for the different detectors used. The dead time map can be calculated pixel-by-pixel from the incoming and outgoing count rate (ICR and OCR) map using the following formula:

$$DT(\%)=\left(1-\frac{ICR-OCR}{ICR}\right)\;x\;100$$ (Eq 1)

S3 Fig shows the incoming (ICR) and outgoing count rate (OCR) map, recorded for each SDD detector (numbered from 0 to 5) of a FRDA fibroblast case ‘DJS’. ICR and OCR map for SDD detector no. 0-1-2 is much lower than for detector no. 3-4-5 due the better alignment of the latter detector pair. The structures observed in the map generally corresponding to the presence of microcrystals, producing elastically scattered photons and secondary (gold, copper) fluorescence from the sample chamber and detector.

### Standard reference material & limits of detection

After optimization of the experimental set-up upon an FRDA fibroblast, the reference material NIST SRM 1577C ‘bovine liver’ was analyzed. NIST SRM 1577C powder contains most of the elements present in single cells and is particularly well-known for its excellent homogeneity on the (sub-) micrometer scale [26]. Another suited reference material for XRF quantification are multi-element thin film standards which can nowadays be produced with nanometer thickness (AXO Dresden GmbH). A total mass of 21.498 *mg* NIST SRM 1577C powder was therefore pressed in a self-supporting (dry) pellet using a die with 13 *mm* diameter pushing rods, resulting in an areal density of 16.20 *mg/cm*^*2*^. Using a scalpel, a *mm*-sized fragment was removed and fixed to a silicon nitride (Si_3_N_4_) membrane. The Si_3_N_4_ wafer was then manually plunge frozen in the Leica™ EM VCM and transferred into the sample chamber under identical conditions.

For investigating the homogeneity of NIST SRM 1577C, two XRF maps were collected: first, a randomly selected area of 2.5 x 2.5 *μm*^*2*^ was scanned in 25 *nm* steps with 100 *ms* dwell time, resulting in an element map of 100x100 points. Second, an XRF map was obtained with size approx. one order of magnitude larger, i.e. 25 x 25 *μm*^*2*^ in 250 *nm* steps with identical dwell time of 100 *ms* was measured. S4 Fig shows the elemental distributions of K, Ca, Fe, Zn and Compton scattering in NIST SRM 1577C using a) 250 *nm* steps and b) 25 *nm* steps. Homogeneous Compton scatter maps of both scans indicate a constant total areal mass over the scanned area. Other elements present in NIST SRM 1577C which are not shown in S4 Fig showed no apparent inhomogeneity. On the microscopic elemental distribution maps (S4 Fig, 0.25 *μm* step size), larger ‘isles’ spanning several tens of microns and smaller areas of approx. 5 *μm* with increased intensity (indicated by arrows) are present. Calcium hot-spots are present with sizes of 1.75 up to 3.25 *μm*. Excellent homogeneity is observed for iron and zinc, albeit with the sporadic presence of some hot-spots. We did however not observe the presence of micrometer sized nuggets containing cupper as reported in another study, scanning a 10 x 10 *μm*^*2*^ area of NIST SRM 1577C, using 200 *nm* steps and 500 *ms* [27]. The nanoscopic element distribution maps (S4 Fig, 25 *nm* step size) exhibits much less heterogeneities for potassium and calcium. For iron and zinc, some more intense areas of approx. 100 *nm* are observed. Note however that this improved homogeneity is observed within a 2.5 x 2.5 *μm*^*2*^ area.

S5 Fig shows the obtained relative limits of detection (LODs, expressed in *ppm*) of NIST SRM 1577C for the elements P, S, Cl, K, Ca, Mn, Fe, Cu, Zn and Rb when using the XRF sum spectrum of the largest XRF map collected (25x25μm^2^, see previous paragraph). LODs were calculated based on: 1) the sum spectrum of all SDD detectors available (no. 0 to 5), 2) half of the detector array providing the better XRF signal (detectors no. 3, 4 and 5) and 3) SDD detector no. 5 only providing optimal iron elemental maps (see earlier). Measurements were performed in ‘high dose’ (HD) mode (2.10^11^ *photons/s*). From the graph, we clearly observe a higher LOD when using a single SDD detector compared to using all detectors, e.g. 8 *ppm* instead of 2 *ppm* for iron, corresponding to a downturn factor of almost 5. Note that this is almost double the theoretical factor of $\sqrt{6}$ (or 2.45) in case all detectors would have equal XRF yield. An improved LOD when using more detectors would plead to use this configuration, nevertheless the iron signal of the fibroblast is completely masked by this configuration as indicated previously in S2 Fig. Using only detector element no. 5, a relative LOD of approx. 8 *ppm* is derived for iron, corresponding to a molar LOD of 140 *μM* or to an absolute LOD of 1 *ag*, equivalent to 12000 iron atoms. For the sake of completeness, LODs (relative, areal, molar, absolute and atomic) for the other detectable elements of biological interest (P, S, Cl, K, Ca, Mn, Fe, Cu and Zn) using detector no. 5 only are provided in S1 Table.

### XRF quantification

#### Data fitting and normalization

Spectral analysis was performed using the AXIL (Analysis of X-ray spectra by Iterative Least squares) [28], including the elements Al, Si, P, S, Cl, K, Ca, Cr, Mn, Fe, Ni, Cu, Zn, Sr and Au, which were fitted using an orthogonal polynomial of 12^th^ order. Batch processing was performed using the MICROXRF2, written in IDL programming language. Raw elemental maps (expressed in *counts* or *cts*) were then normalized to 1*s* measuring time, incoming beam intensity ‘*I*_0_’ (*a*.*u*.) and dead time ‘*DT*’ (in *%*). In this manner, normalized element maps are obtained (expressed in *cts/s*). As incoming beam intensity detector was not properly obtained during our measurements, elemental maps were normalized to the transmitted beam intensity ‘*I*_*t*_’ since absorption effects of the cell on the transmitted intensity map could be considered negligible. For additional information concerning the normalization procedure we refer to the Materials and Methods section in another work [25].

#### Determination of ice layer thickness

After cryofixation and blotting—an action which removes the excess of water before submersion into a cryogen—an ice layer generally remains covering the fibroblasts. Since the whole procedure can be effected by the human hand or robotically, the ice layer thickness varies with the position of the cell on the membrane (and also from membrane to membrane). For accurate calculation of the mass fractions of the lower atomic number elements such as P, S, Cl, K, and Ca, where absorption of the lower energetic fluorescent X-rays plays a significant role, the thickness of the ice layer covering the cells is an important quantity which needs to be determined. For calculating the ice layer thickness d_ice_ for which 50% of the original fluorescence of a probed element *i* remains, the following relation must apply:

12If,ice(i)=If,ice(i)e−χice(i)(ρd)ice (Eq 2)

which can be simplified to:

$$d_{ice}=\frac{ln(2)}{{\left(\chi \rho \right)}_{ice}}$$ (Eq 3)

S2 Table (left side) summarizes the ice layer thickness for which 10, 50 and 99% of the K_α_ fluorescent photons are absorbed (*T*_*90%*_, *T*_*50%*_ and *T*_*1%*_). For phosphorus, sulphur and chlorine, 50% of the original fluorescent intensity is already absorbed by a thin ice layer of only 2.9, 4.4 and 6.3 *μm*; 99% of the fluorescence of these elements is absorbed by an ice layer of 20, 29 and 42 *μm* thick, respectively. Also for potassium and calcium, 50% of the K_α_ fluorescence is absorbed after 12 and 17 *μm* of ice, respectively. Manganese, iron and zinc having higher K_α_ fluorescent energies are less problematic: 90% of the fluorescence still penetrates through an ice layer thickness of 10, 13 and 32 *μm*, respectively.

Two methods were examined for calculation of the ice layer thickness *d*_*ice*_ above the single fibroblasts, i.e. using the total X-ray transmission (I_0_/I_t_) on the one hand and by using the K-K_α_/K_β_ ratio on the other hand. For the ‘I_0_/I_t_’ method, the X-ray beam intensity was measured using a diode with the sample in the X-ray focus, resulting in an intensity ‘*I*_*sample*_’ measured; thereafter without the sample in the X-ray focus, providing an intensity ‘*I*_*0*_’. As the primary X-ray beam is attenuated both by the silicon nitride membrane and the ice layer on it, the following equation applies according to Lambert-Beer’s attenuation law:

$$I=I_{0}(e^{-\mu_{0}(ice){\left(\rho d\right)}_{ice}})\;(e^{-\mu_{0}({Si}_{3}N_{4}){\left(\rho d\right)}_{{Si}_{3}N_{4}}})$$ (Eq 4)

which provides after rearrangement the following measure for the ice layer thickness d_ice_:

dice=[ln⁡(I0Isample)−μ0(Si3N4)(ρd)Si3N4](μ0,iceρice) (Eq 5)

Since the used Si_3_N_4_ membranes have a thickness of 500 *nm* (or 500 .10^−7^ *cm*), a density of *3*.*4 g/cm*^*3*^ and a total X-ray cross section of 4.583 *cm*^*2*^*/g* at 17.1 *keV* and the total X-ray cross-section ice is 1.186 *cm*^*2*^*/g* at 17.1 *keV*, the following formula is obtained for determining the ice layer thickness *T*_*ice*_ (in *cm*):

$$d_{ice}=\left[ln(\frac{I_{0}}{I_{sample}})-3.1531.10^{-4}\right]/1.186$$ (Eq 6)

where *I*_0_ represents the total measured XRF intensity while having the sample out of the beam, *I*_*ice*_ the total XRF intensity of the sample within the beam, ${µ}_{H_{2}0}$ and ${µ}_{{Si}_{3}N_{4}}$ the total X-ray cross-section of water and silicon nitride, respectively. 500.10^−7^ refers to the 500 *nm* thickness of the Si_3_N_4_ membrane. This method is however has inaccuracies as: 1) it takes into account non-present absorption from ice behind the wafer 2) the measurement in only performed at one specific position on the cell and 3) it considers absorption along the direct beam path which is different from the fluorescence path towards the detector. Therefore, a second more indirect way was used to determine the ice layer thickness by using the K-K_α_/K_β_ signal: due to the fact that the K_α_ fluorescent is lower in energy than the K_β_ radiation and K is richly present in all cells, the ratio *R* of both fluorescent intensities can be used to calculate the thickness of the ice layer through which they travel. When no absorption occurs, *R* can be calculated from the X-ray radiative rates of K-K_α_ and K-K_β_ as follows:

$$R=\frac{I(K-K_{\alpha ,1})}{I(K-K_{\beta ,1})}$$ (Eq 7)

Therefore, the following relation applies for the K-K_α_ and K-K_β_ ratio R_0_ for the case without absorption occurring and the case R with absorption occurring through an ice layer:

$$R=R_{0}\;e^{-(\mu_{{K-K}_{\alpha ,1}}-\mu_{{K-K}_{\beta ,1}}){\left(\varphi d\right)}_{H_{2}O}}$$ (Eq 8)

After rearrangement, the following equation for the ice layer thickness (in g/*cm*^*2*^) is obtained:

$${\left(\varphi d\right)}_{ice}=ln(\frac{R}{R_{0}})/-(\mu_{K-K_{\alpha ,1}}-\mu_{{K-K}_{\beta ,1}})$$ (Eq 9)

By assuming that the density of the ice formed after plunge freezing is *φ*_*ice*_, the total distance *d*′_*ice*_ that the X-ray photons travel through the ice is given by:

$${d\prime }_{ice}={\left(\varphi d\right)}_{ice}/\varphi_{ice}$$ (Eq 10)

If the detector angle between the sample surface and detector amounts α radians, then the true ice layer thickness *d*_*ice*_ covering the cells can be calculated according to the following formula:

$$d_{ice}=\sin\left(\alpha \right).{d\prime }_{ice}$$ (Eq 11)

Note that this method is a more accurate measure for the ice layer thickness as it only accounts for absorption in the ice layer through which the fluorescent photons travel. The first method discussed can also take into account ice behind the sample not responsible for absorption. Therefore, values for ice layer thickness obtained by the first method should be systematically higher than the ice layer thickness determined using the K-K_α_/K_β_ method.

By using the K-K_α_/K_β_ ratio, we found an ice layer thickness of approx. 7.6 *μm* and 4.0 *μm* covering the control fibroblast from case ‘PN’ and FRDA fibroblast from case ‘DJS’, respectively. The calculated ice thickness obtained using X-ray transmission values (I_0_/I_t_ method) were generally higher: 14 *μm* relative to 7.6 *μm* for the control fibroblast case ‘SL’ and 50 *μm* relative to 4.0 *μm* for the FRDA fibroblast case ‘DJS’. This is most probably due to the additional amount of ice behind the Si_3_N_4_ membrane which is also taken into account when using the ‘I_0_/I_t_’ method. The percentage of absorbed K_α_ fluorescence for a specific element using the calculated ice thickness of the control and FRDA fibroblast using both methods (‘I_0_/I_t_’ and ‘K-K_α_/K_β_ ratio’) is shown in Table 2. The higher ice thicknesses obtained for the ‘I_0_/I_t_’ method cause absorption up to 90%, even for calcium; for the ‘K-K_α_/K_β_ ratio’ method this is only a mere 20%.

### Determination elemental mass fractions within single, cryofrozen cells.

For determining the fluorescence radiation for a particular trace element *i* emanated from a homogeneous cell layer, the Fundamental Parameter equation applies

$$I_{cell(i)}=I_{0}\;G\;\omega_{i,cell}\;Q_{i,K_{\alpha }}\frac{1-e^{-\chi_{cell(i)}\;{\left(\rho d\right)}_{cell(i)}}}{\chi_{cell(i)}}$$ (Eq 12)

for which I_0_ is the incoming photon flux, *G* the geometry factor, ω_i_ the mass fraction of element *i*, (*ρd)*_*cell*_ the areal density of the cell, *Q*_*i*, *Kα*_ the production cross-section of the element *i* in the cell at the incoming photon energy. The term χ_cell(i)_ is a factor which corrects for absorption effects of exciting and fluorescent X-rays within the frozen cell layer:

$$\chi_{cell(i)}=\left(\frac{\mu_{0,cell}}{\sin\alpha }+\frac{\mu_{i,cell}}{\sin\beta }\right)$$ (Eq 13)

where α is the angle between the incoming X-ray beam with the sample surface and β the angle between the detector and sample surface. *μ*_*0*,*cell*_ is the total X-ray cross-section of the cell layer at the incoming photon energy E_0._ The total X-ray cross-section of the virtual cell layer for a particular element *i* is represented by *μ*_*i*,*cell*_. Both quantities are defined by the mass fraction *ω*_*i*_ of a particular element *i* multiplied by the photo-electric X-ray cross section τ_*i*_ at the incoming excitation energy *E*_*0*_ or fluorescent energy *E*_*f*,_, respectively:

$$\mu_{0,cell}=\sum_{i}\omega_{i}\tau_{i,E_{0}}$$ (Eq 14)

$$\mu_{i,cell}=\sum_{i}\omega_{i}\tau_{i,E_{f}}$$ (Eq 15)

All photo-electric and total X-ray cross sections were obtained from the Xraylib database [29, 30] called from IDL (Interactive Data Language).

In our experiment cells are additionally covered by an ice layer, absorbing its original fluorescence I_f,orig_ according to the law of Lambert-Beer:

$$I_{cell,ice\;(i)}=I_{cell(i)}\;e^{-\chi_{ice,i}{\;(\rho d)}_{ice}}$$ (Eq 16)

Hereby is (*ρd*)_*ice*_ the areal mass of the deposited ice layer above the cell, expressed in *g/cm*^*2*^. The ice layer thickness above the cell *d*_*ice*_ can now be determined as discussed in previous section using Eq 6 (I_0_/I_t_ method) or Eqs 9–11 (K-K_α_/K_β_ ratio method). By assuming that ρ_ice_ = 1*g/cm*^*3*^, an ice layer thickness of x *μm* is equivalent to an areal mass of x 10^−4^ *g/cm*^*2*^. The factor χ_ice,i_ in Eq 16 is composed of μ_0,ice_ and μ_i, ice_ in analogy to Eq 13. However χ_ice(i)_ is easier to calculate than χ_cell(i)_ due to the divalent composition of ice:

μ0,ice=218τH(E0)+1618τO(E0) (Eq 17)

μi,ice=218τH(Ef)+1618τO(Ef) (Eq 18)

By combining Eq 12 and Eq 16, the detected fluorescent intensity from an element *i* from the cell covered by an ice layer is given by:

$$I_{cell,i}=I_{0}\;G\;\omega_{i,cell}\;Q_{i,K_{\alpha }}\left(\frac{1-e^{-\chi_{cell(i)}\;{\left(\rho d\right)}_{cell(i)}}}{\chi_{cell(i)}}\right)\;(e^{-\chi_{i,ice}\;{\left(\rho d\right)}_{ice}})$$ (Eq 19)

As we can consider the cell as a thin, i.e. linear absorption in function of thickness, the first term in brackets in Eq 19 can be simplified, providing the following equation:

$$I_{cell,i}=I_{0}\;G\;\omega_{i,cell}\;Q_{i,K_{\alpha }}\;{\left(\rho d\right)}_{cell}\;e^{-\chi_{i,ice}\;{\left(\rho d\right)}_{ice}}$$ (Eq 20)

For quantification of the elemental maps, a calibration standard, i.e. a thin pellet of NIST SRM 1577C with an areal density (*ρd)*_*SRM*_ of 16.20 *mg/cm*^*2*^ was measured under identical conditions as the fibroblast cells (see also Materials and Methods, ‘Standard Reference Materials & Limits of Detection’). For the SRM, the fundamental parameter equation applies as well:

$$I_{SRM(i)}=I_{0}\;G\;\omega_{i,SRM}\;Q_{i,K_{\alpha }}\frac{1-e^{-\chi_{SRM(i)}{\;(\rho d)}_{SRM(i)}}}{\chi_{SRM(i)}}$$ (Eq 21)

S6 Fig shows both *μ*_0_ at 17.1 *keV* and *μ*_*i*_ for the elements P, S, Cl, K, Ca, Mn, Fe, Ni, Cu and Zn for an ice matrix (blue curve) and for NIST SRM 1577C ‘bovine liver’ (red curve).

By dividing Eq 20 and Eq 21 by each other, *I*_*0*_, *G* and $Q_{i,K_{\alpha }}$ are cancelled out. Since in Eq.4.7 *ω*_*i*,*cell*_(*ρd*)_*cell*_ actually represents the areal concentration (in *g/cm*^*2*^) of a certain element *i* in the cell, we obtain after rearrangement that:

$$c_{areal,cell}(\frac{g}{{cm}^{2}})=\frac{I_{cell,i}}{I_{SRM,i}}\;\omega_{i}\;\left(\frac{1-e^{-\chi_{SRM(i)}\;{\left(\rho d\right)}_{SRM(i)}}}{\chi_{SRM(i)}}\right)\;{\left(e^{-\chi_{i,ice}\;{\left(\rho d\right)}_{ice}}\right)}^{-1}$$ (Eq 22)

In which the first term between brackets symbolizes the self-absorption correction factor for the SRM while the second term stands for the absorption correction due to the ice layer. S7 Fig. shows both correction factors between brackets separately and their product.

From the areal concentration maps (expressed in e.g. in *μg/cm*^*2*^), the mass fraction maps can be obtained assuming a mean cell thickness of 10 *μm*, resulting in a total areal mass of the cells of 1 *mg/cm*^*2*^. Dividing the areal concentrations maps by the total estimated areal mass of the cells finally provides the mass fractions maps in *ppm*:

$$w_{i,cell}\;\left(\frac{µg}{g}or\;ppm\right)=\frac{c_{areal,cell}(\frac{µg}{cm^{2}})}{0.001\;g/cm²}$$ (Eq 23)

After conversion of the elemental maps, masks were created selecting only the pixels belonging to the entire cell, cytoplasm, nucleus or background. To obtain accurate quantitative values, several measures were taken: (1) subtraction of background mass fraction from the cytoplasm mass fractions (2) cluster areas of cell and cytoplasm were not taken perfectly adjacent since stray fluorescence of the cell can contribute to the background and (3) isolation and removal of XRF point spectra from ‘hot spots’ within the cell identified as inorganic (dust) particles and therefore artificially increasing the element concentration. Masks were then multiplied with the quantified maps of each element from which a mean concentration value could be obtained. Due to the rather large background for the elements Fe, Cu and Au induced by fluorescence from the sample holder and vacuum chamber, the mean concentration value of the background was subtracted from the mean cell concentration values. A flowchart illustrating the entire quantification workflow for an FRDA fibroblast raw elemental map is provided in S8 Fig.

## Summary and conclusions

Trace level elemental distributions within cryogenically frozen (-150°C) fibroblasts from control-disease and Friedreich’s ataxia (FRDA) patients were obtained using synchrotron radiation (SR) based nanoscopic hard X-ray fluorescence (XRF) using an X-ray nanobeam with unprecedented spatial resolution of 20 *nm*, currently a world’s first. Human fibroblast cells were cultured on the synchrotron site, after which the support membranes were automatically plunge frozen, mounted and transferred to the cryocooled sample stage of the ID16A-NI ‘Nano-imaging’ end-station, all in an uninterrupted cryogenic workflow. Although we did observe some radiation damage effects upon the cryogenically frozen fibroblasts such as slow freeze-drying or ablation of thinner micrometer regions, nanoscopic XRF scanning under cryogenic conditions generally maintained the elemental distributions of fibroblast cells for more than several hours, even under extremely high flux conditions of 2x10^11^ *photons/s*. Owing to this high flux, relative limits of detection (LODs) for our experiment reached 7.6 *ppm* in scanning mode for iron, equivalent to absolute LODs of 140 *nM* or approx. 12.000 atoms. LODs were generally higher compared to non-cryogenic mode due to the higher production of scattered X-ray photons for frozen cells, increasing the background signal and secondary fluorescence from sample holder and vacuum chamber. Elemental maps of the fibroblasts also showed the virtual presence of copper, nickel and gold confined within microscopic areas, which were caused by the presence of microcrystals in the shock-frozen fibroblasts—supposedly containing amorphous ice only—diffracting incoming X-ray photons.

For accurate quantification of the (FRDA) fibroblast elemental maps, estimation of the ice layer thickness turned out to be critical, especially for lighter elements such as P, S, Cl, K and Ca. By using the K_α_/K_β_ fluorescence ratio of potassium, the ice layer thickness covering each fibroblast could be determined, typically ranging from a few *μm* up to 100 *μm*. Ice layer thickness varied strongly with the blotted sample and position. The K_α_/K_β_ fluorescence ratio of potassium was used for estimating the thickness of the ice layer covering the cells because of its prominent presence in the fibroblasts. Background-corrected mass fraction elemental maps for the elements P, S, Cl, K, Ca, Fe and Zn were finally determined for the control and FRDA fibroblast using the Fundamental Parameter (FP) quantification method. Within high resolution (55 nm) XRF maps, iron-rich hot-spot areas were detected in the cytoplasm of both a control and FRDA fibroblast, which were estimated to have a concentration of approx. 210 *ppm*. Highly pronounced iron enrichments as observed in FRDA fibroblasts by Prussian blue staining were however not found, which may be related to the fact that fibroblasts were kept in culture for several cycles, imposing an unnatural environment compared to *in vivo* conditions. As metal metabolism of fibroblasts may already change after a few cell cycles, immediate XRF analysis after skin biopsy is recommended. Another reason could be that given the limited sample throughput of SR based XRF, only FRDA fibroblasts without iron accumulation were scanned; in this respect pre-selection of the cells of interest using fluorescence-activated cell sorting (FACS) would be a valuable approach. From medium resolution (200 nm) XRF maps, mean mass fractions of the cytoplasm within 5 control and 5 FRDA fibroblasts were calculated. Also here, no significant cytosolic iron increase for FRDA fibroblasts was observed. However, provided the multi-element character of SR based XRF, we detected a significant decrease in zinc for the FRDA fibroblasts, which may be related to impaired homeostasis of this/these element(s) for FRDA. While Zn^2+^ is not redox active under physiological conditions, zinc deficiency causes increased oxidative damage to DNA, proteins, and lipids, indicating an indirect antioxidant role for zinc as a required element in CuZnSOD or MnSOD superoxide dismutase (SOD) [31].

We highlight synchrotron radiation based nanoscopic XRF analysis under cryogenic conditions as a new method of choice for life science investigations requiring the ultimate trace level subcellular elemental distribution or aiming at a quantitative study of several cells close to the *in vivo* state. Owing to the current availability of nanoscopic XRF analysis under cryogenic conditions, the debate on the validity of elemental distributions obtained on chemically fixed cells–prone to contamination and metal relocation- becomes largely unnecessary. Similar to XRF analysis under ambient temperature, a wide range of biological elements can be probed simultaneously (P, S, Cl, K, Ca, Mn, Fe, Co, Ni, Cu, Zn, Se and Br), sometimes even with better sensitivity for the lighter elements due to the required vacuum conditions in cryogenic mode. In contrast to staining reagents or visible light fluorescent probes, X-ray fluorescence allows to measure the sample directly, without the use of any chemicals. Future challenges of nanoscopic XRF imaging under cryogenic conditions include improving sensitivity by better shielding of the detector array from the surrounding sample environment or by installation of a dual detector setup, providing natural shielding against background fluorescence. The observed presence of microcrystals within the vitreous ice matrix causing diffraction, secondary fluorescence and increased background could be overcome by optimization of the shock-freezing methods preventing the formation of these crystals. More accurate estimation of the ice layer thickness should lead to more reliable quantification for lower atomic number elements such as phosphorus, sulphur, chlorine and potassium. Another possibility is to produce a well-defined (or even constant) ice layer covering the cells by more reproducible blotting procedures or the fabrication of cryosections [32]. Due to the planned upgrades of different 3^rd^ generation synchrotron radiation sources (ESRF-EBS and PETRA IV), an increase in brilliance of 10 to 100 times is to be expected. This will result in up to 10 times more sensitive nanoscopic XRF imaging in the same time frame currently used, which will enable visualization of (ultra) trace elements in single cells, e.g. nickel and selenium. On the other hand, when settling with the current sensitivity, single cells will be scanned in a few minutes rather than several hours.

## Supporting information

S1 Fig Elemental maps of Si, P, S, Cl, K, Ca, Cr, Mn, Fe, Ni, Cu, Zn, Sr, Au and Compton/Rayleigh scatter of FRDA fibroblast case ‘DJS’ obtained for each SDD detector (0, 1, 2, 3, 4 and 5) separately. The total scanned area covers 44.9 x 32.7 *μm*^*2*^. Elemental maps were obtained at ‘High dose’, using a step size of 55 *nm* and a dwell time of 55 *ms*. Elemental maps showing clear fibroblast structure are indicated using green rectangles. Virtual presence of Ni, Cu and Au through secondary fluorescence is indicated with red rectangles.
(TIF)

S2 Fig Elemental maps of iron and potassium of FRDA fibroblast case ‘DJS’ for detector no. 3, 4 and 5. The total scanned area covers 44.9 x 32.7 *μm*^*2*^. Elemental maps were obtained at ‘High dose’, using a step size of 55 *nm* and a dwell time of 55 *ms*. Cell border and nucleus are indicated on the iron elemental map of detector no. 5 for clarity using a dashed and full black line, respectively. Within the same elemental map, the presence of iron containing hot-spots is indicated with a red striped circle.
(TIF)

S3 Fig Incoming count rate (ICR) and outgoing count rate (OCR) map from scan on FRDA fibroblast case ‘DJS’ for detector 0,1,2,3,4 and 5. For each detector, the corresponding dead time map is shown.
(TIF)

S4 Fig Elemental distributions of K, Ca, Fe, Zn and Compton scattering in NIST SRM 1577C ‘bovine liver’. Elemental maps were obtained under cryogenic conditions at ID16A-NI ‘Nano-imaging’ beamline at the ESRF in Grenoble (France). ‘High dose’ mode, pixel size: 250 *nm* (a) and 25 *nm* (b), 100 *ms* dwell time.
(TIF)

S5 Fig Relative LODs obtained for NIST SRM 1577C ‘bovine liver’ at beamline ID16A-NI for different number of detectors used. LODs were calculated in ‘High Dose’ mode. Relative LOD is calculated for integrated signal from all 6 detector (blue), integrated detector signal from detectors no. 3-4-5 (orange) and from detector no. 5 only (grey). Colored curves are added to guide the eye.
(TIF)

S6 Fig Total absorption cross-section μ_i_ of pure ice (blue curve) and within NIST SRM 1577C ‘bovine liver’ (red curve). Results are expressed in *cm*^*2*^*/g*. A curve was added to ‘guide the eye’. The total absorption cross-section of the incoming photon energy (17 *keV*) within both matrices (ice, bovine liver) was determined as well.
(TIF)

S7 Fig Correction factors when determining the elemental concentrations within a single cell covered by an ice layer using the Fundamental Parameter equation and NIST SRM 1577C as calibration standard. Correction factor for self-absorption in NIST SRM 1577C (blue), in ice (blue) and total absorption correction factor (green).
(TIF)

S8 Fig Quantification workflow illustrated for a XRF map of a human FRDA fibroblast case ‘DJS’. From the incoming (ICR) and outgoing count rate map (OCR), the dead time percentage map is determined (in %). Together with the *I*_*0*_ map (in *a*.*u*.) and the raw element intensity maps (in *cts*), normalized element intensity maps are produced (in *cts/s*). Using the Fundamental Parameter equation, the normalized element maps are converted into concentration maps (in *ppm* or in *μg/cm*^*2*^). Absorption effects in NIST SRM 1577C and in the ice layer covering the cells are corrected for. Background is subtracted from the entire concentration map. Cluster maps can then be generated containing regions of interest (ROIs), such as entire cell, cytoplasm and background, omitting hot-spots. In combination with the quantitative element distributions, background corrected mean concentrations of ROIs are obtained.
(TIF)

S1 Table Relative, areal, molar, absolute and atomic LODs obtained at beamline ID16A-NI for P, S, Cl, K, Ca, Mn, Fe, Cu and Zn in NIST SRM 1577C ‘bovine liver’. LODs were calculated in ‘high dose’ mode. Only a single detector channel was used (XIA05).
(XLSX)

S2 Table Calculation of ice layer thicknesses and K_α_ fluorescence absorbed. Ice layer thickness for which 10, 50 and 99% of the fluorescent photons of the elements P, S, Cl, K, Mn, Fe and Zn are absorbed (left main column). Percentage of absorbed K_α_ fluorescence for a specific element using the calculated ice thickness of the control and FRDA fibroblast using the ‘I_0_/I_t_’ and ‘K-K_α_/K_β_ ratio’ method (right main column).
(XLSX)
