# Correction: Nanoscopic X-ray fluorescence imaging and quantification of intracellular key-elements in cryofrozen Friedreich’s ataxia fibroblasts

**DOI:** 10.1371/journal.pone.0194850

**Published:** 2018-04-30

**Authors:** The PLOS ONE Staff

Fig 3 appears incorrectly. The authors have provided a corrected version here. The publisher apologises for the error.

**Fig 3:**
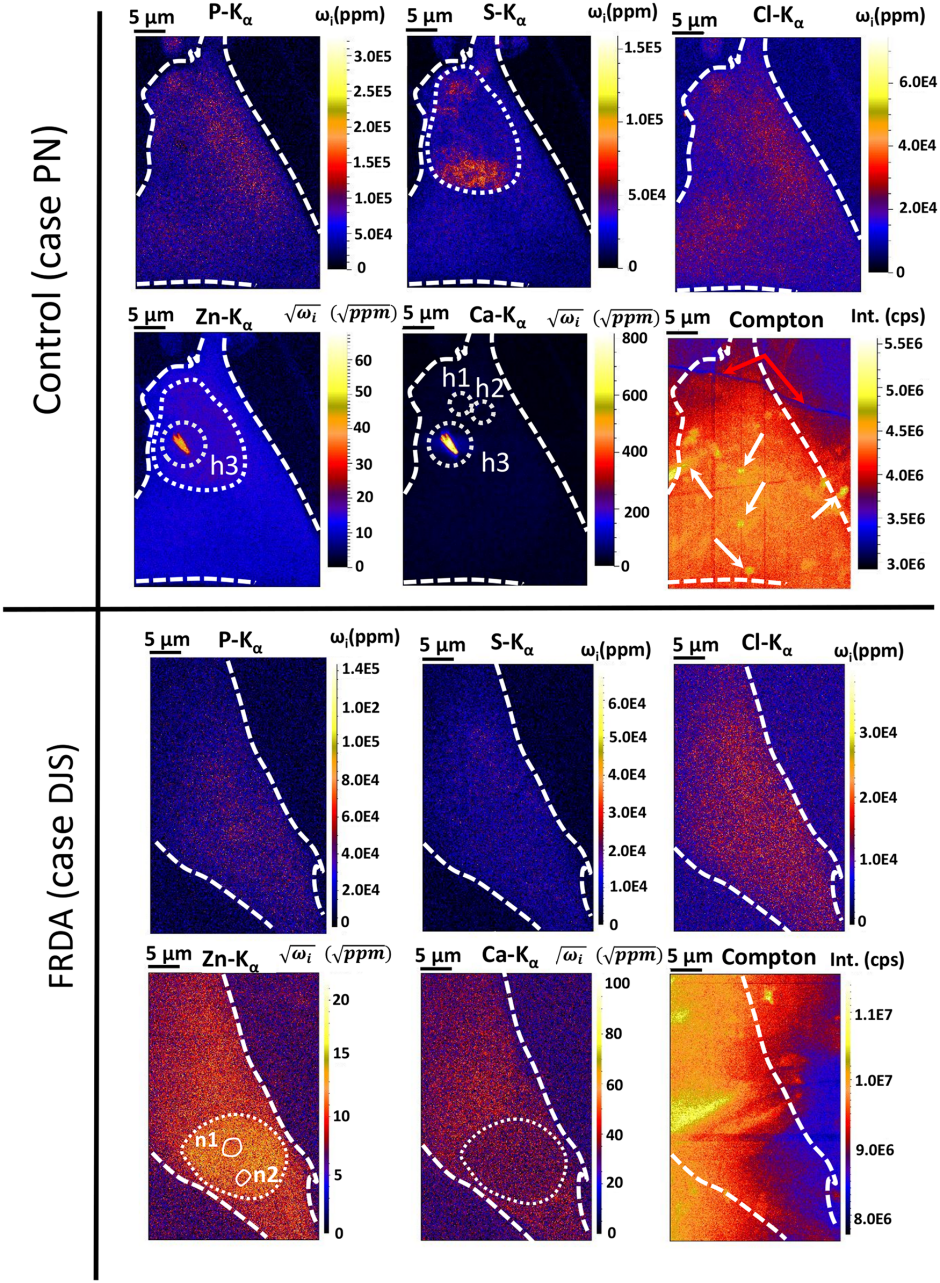
Elemental distribution of P, S, Cl, Ca, Zn and Compton scatter within control fibroblast case ‘PN’ (upper row) and FRDA fibroblast case ‘DJS’ (lower row). Experimental conditions: see legend of Fig 2. White dashed circle indicates the nucleus border. n1-2 indicate the presence of nucleoli.
