# Supplementary figures and images for: Nanoscopic X-ray fluorescence imaging and quantification of intracellular key-elements in cryofrozen Friedreich’s ataxia fibroblasts
Source: PLoS One. 2018 Jan 17;13(1):e0190495. doi: 10.1371/journal.pone.0190495 (PMC5771581; doi:10.1371/journal.pone.0190495)

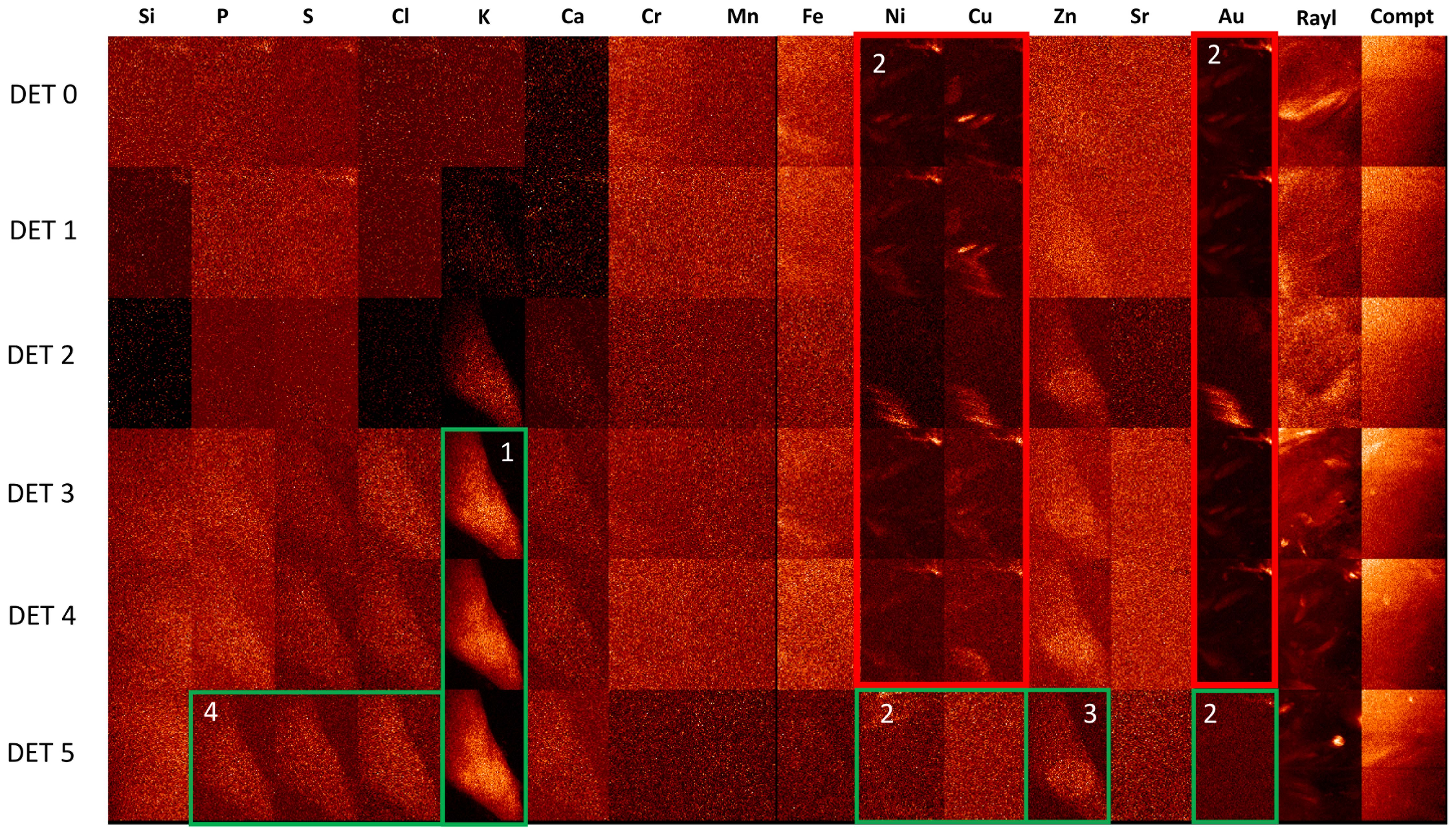

Supplement: S1 Fig — The total scanned area covers 44.9 x 32.7 μm2. Elemental maps were obtained at ‘High dose’, using a step size of 55 nm and a dwell time of 55 ms. Elemental maps showing clear fibroblast structure are indicated using green rectangles. Virtual presence of Ni, Cu and Au through secondary fluorescence is indicated with red rectangles. (TIF) [file pone.0190495.s001.tif]

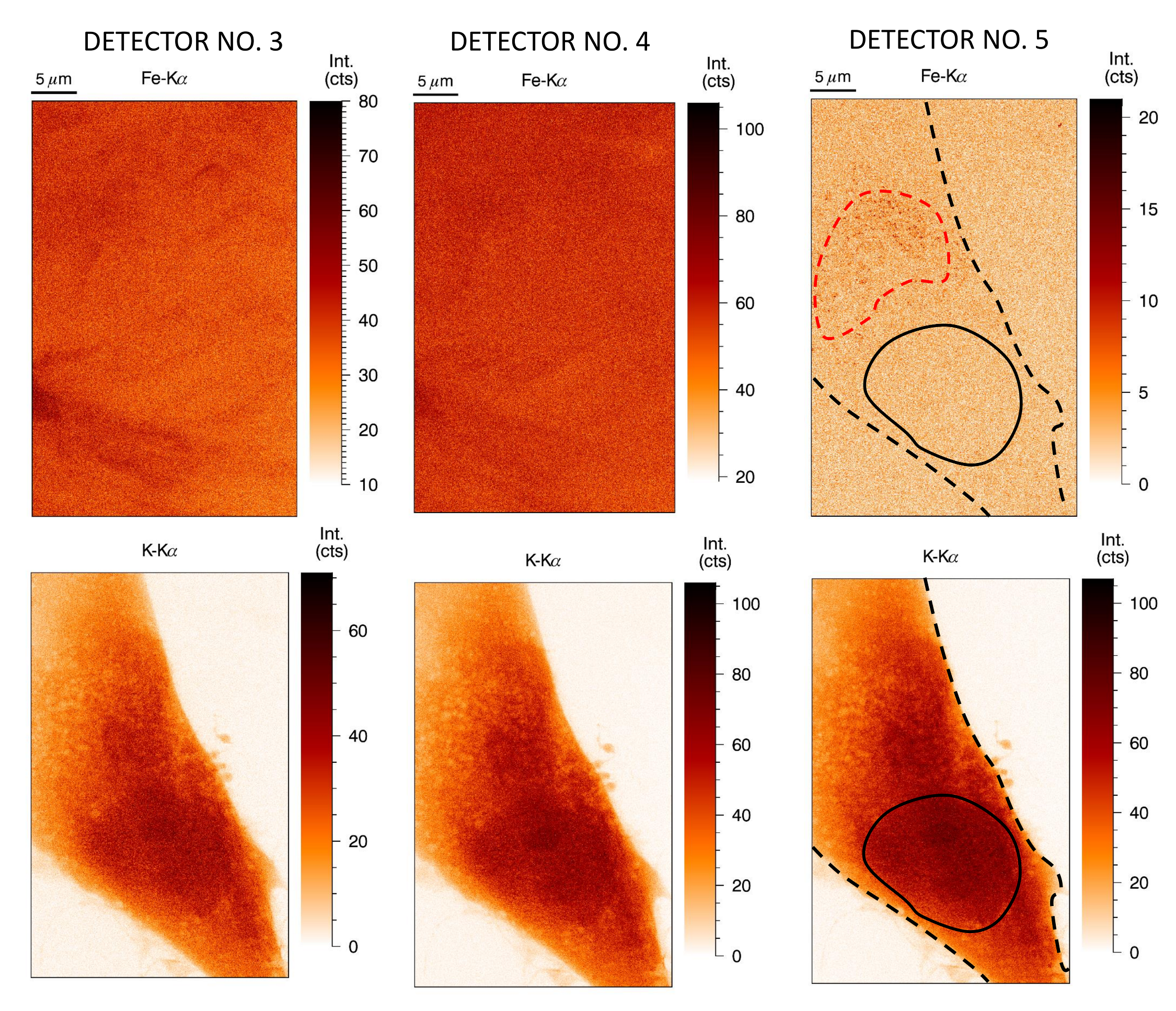

Supplement: S2 Fig — The total scanned area covers 44.9 x 32.7 μm2. Elemental maps were obtained at ‘High dose’, using a step size of 55 nm and a dwell time of 55 ms. Cell border and nucleus are indicated on the iron elemental map of detector no. 5 for clarity using a dashed and full black line, respectively. Within the same elemental map, the presence of iron containing hot-spots is indicated with a red striped circle. (TIF) [file pone.0190495.s002.tif]

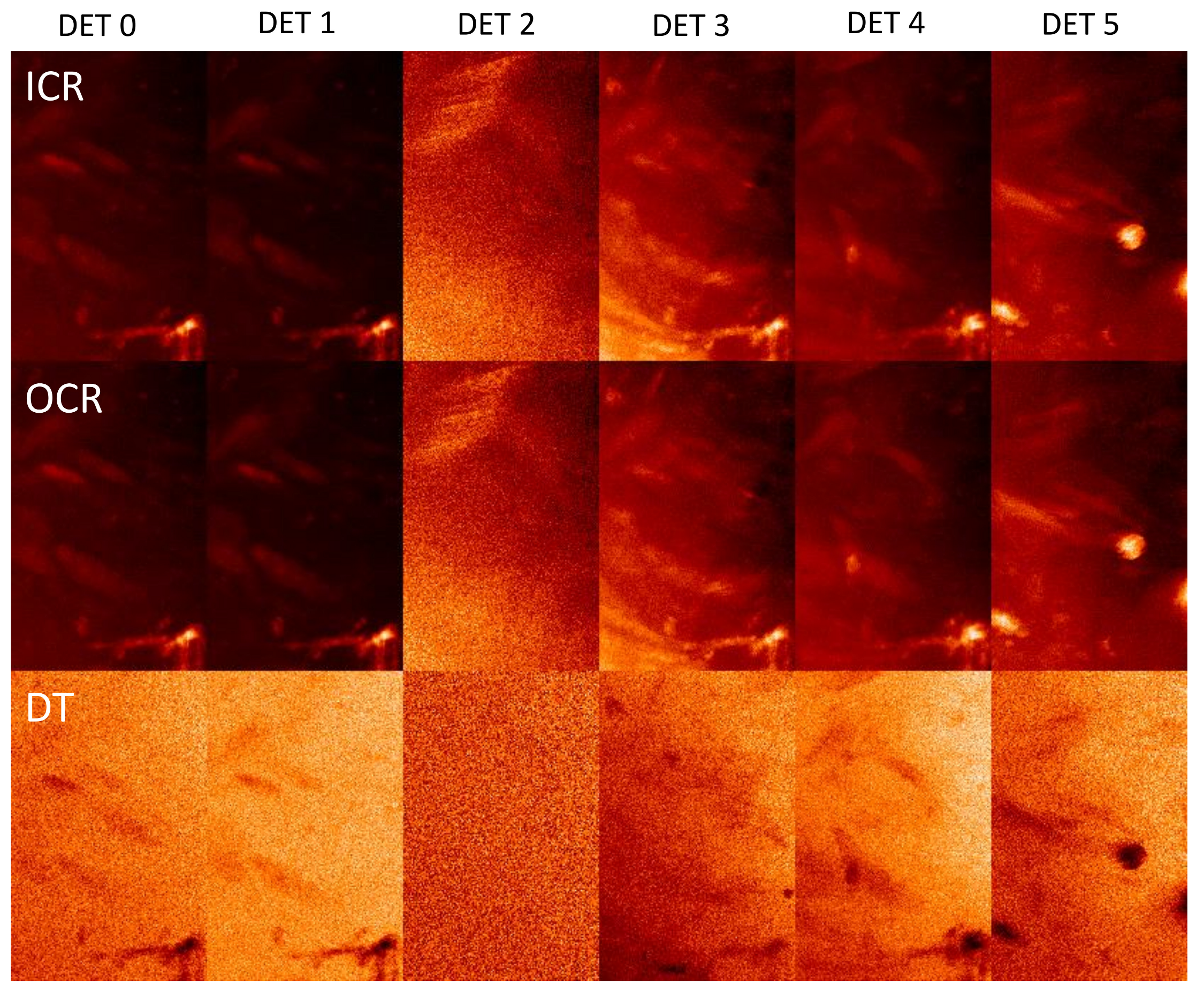

Supplement: S3 Fig — For each detector, the corresponding dead time map is shown. (TIF) [file pone.0190495.s003.tif]

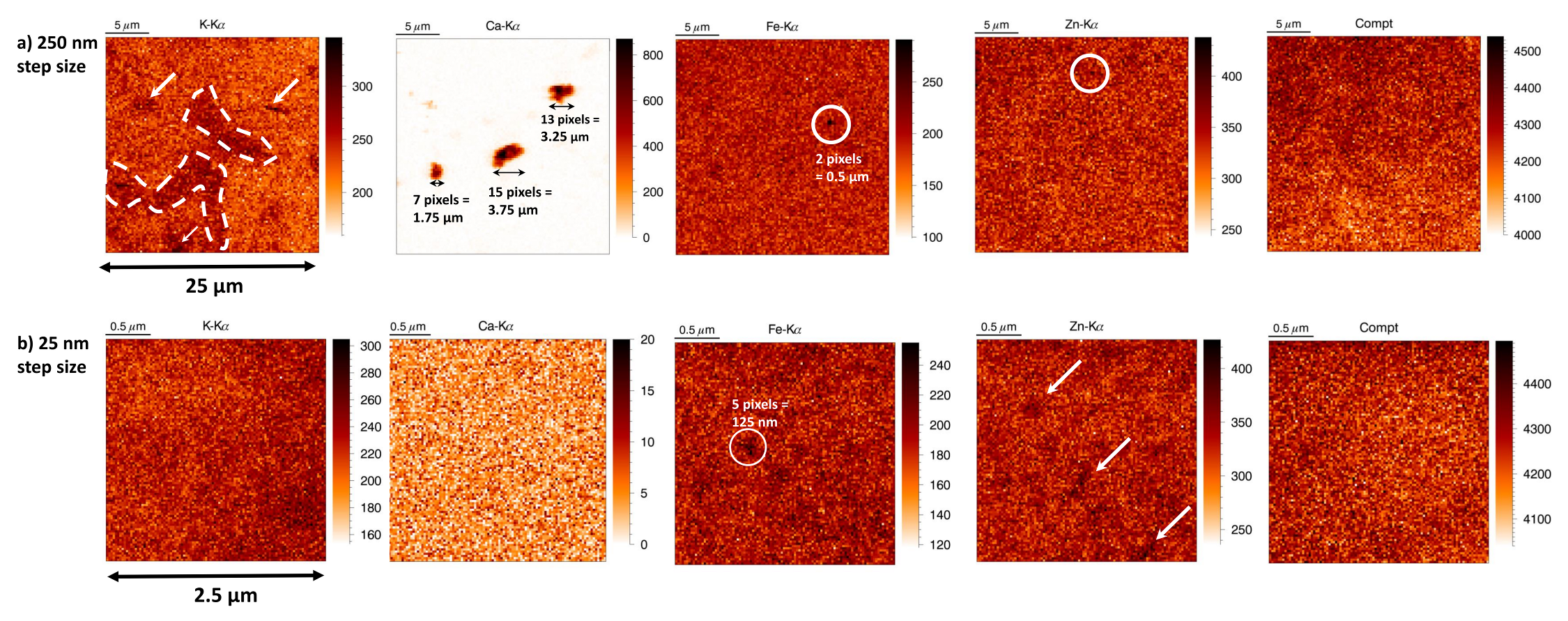

Supplement: S4 Fig — Elemental maps were obtained under cryogenic conditions at ID16A-NI ‘Nano-imaging’ beamline at the ESRF in Grenoble (France). ‘High dose’ mode, pixel size: 250 nm (a) and 25 nm (b), 100 ms dwell time. (TIF) [file pone.0190495.s004.tif]

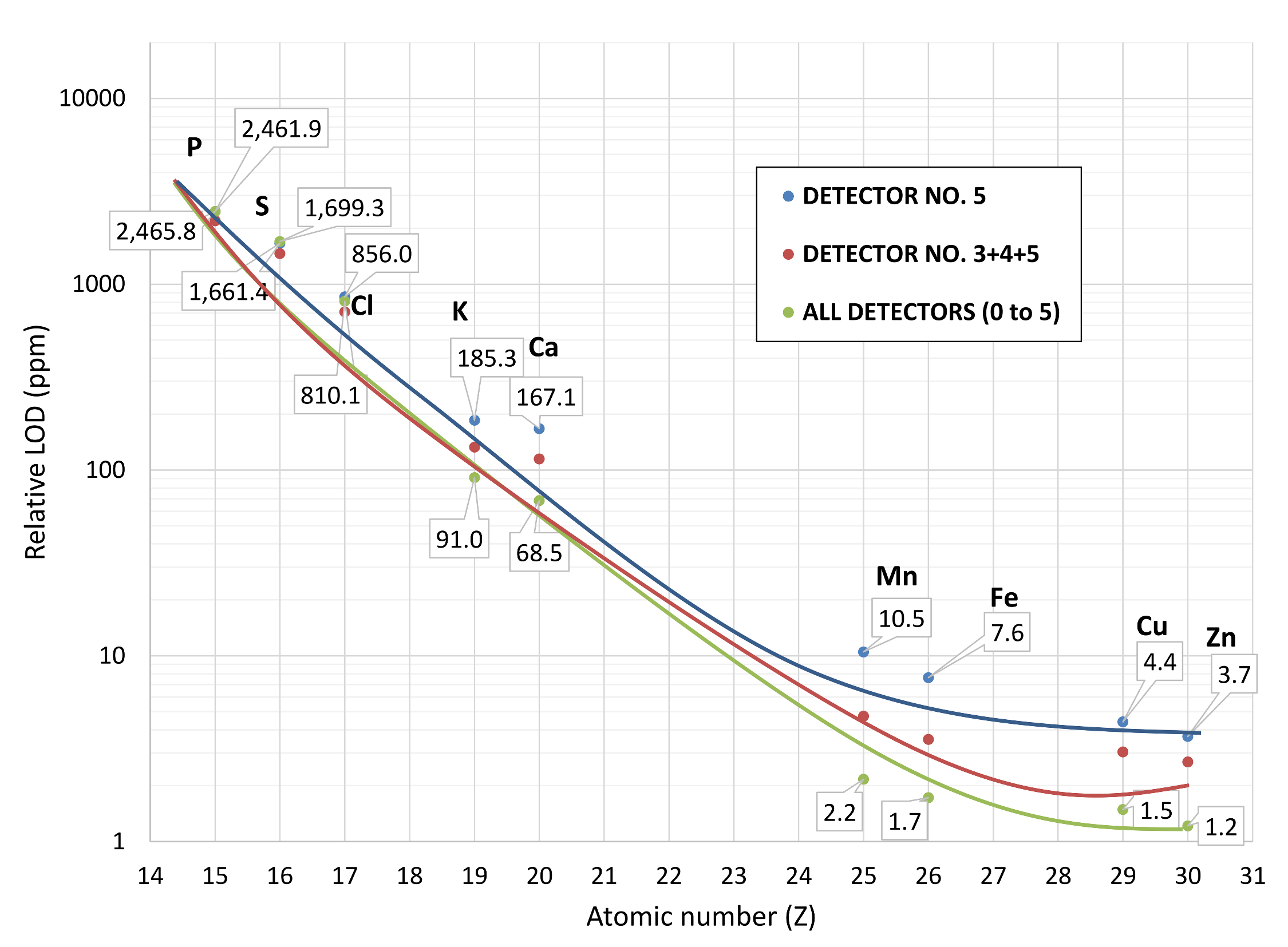

Supplement: S5 Fig — LODs were calculated in ‘High Dose’ mode. Relative LOD is calculated for integrated signal from all 6 detector (blue), integrated detector signal from detectors no. 3-4-5 (orange) and from detector no. 5 only (grey). Colored curves are added to guide the eye. (TIF) [file pone.0190495.s005.tif]

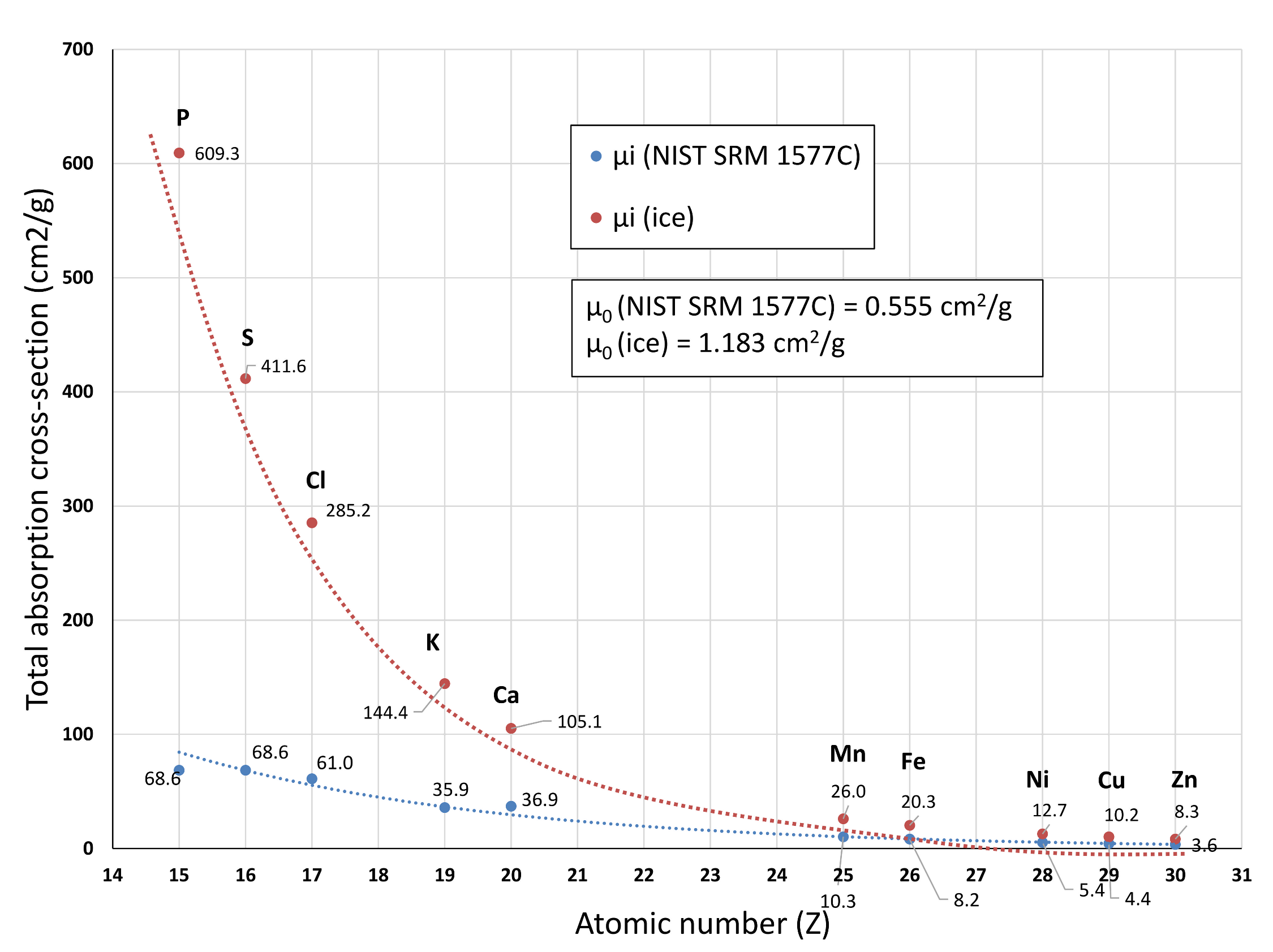

Supplement: S6 Fig — Results are expressed in cm2/g. A curve was added to ‘guide the eye’. The total absorption cross-section of the incoming photon energy (17 keV) within both matrices (ice, bovine liver) was determined as well. (TIF) [file pone.0190495.s006.tif]

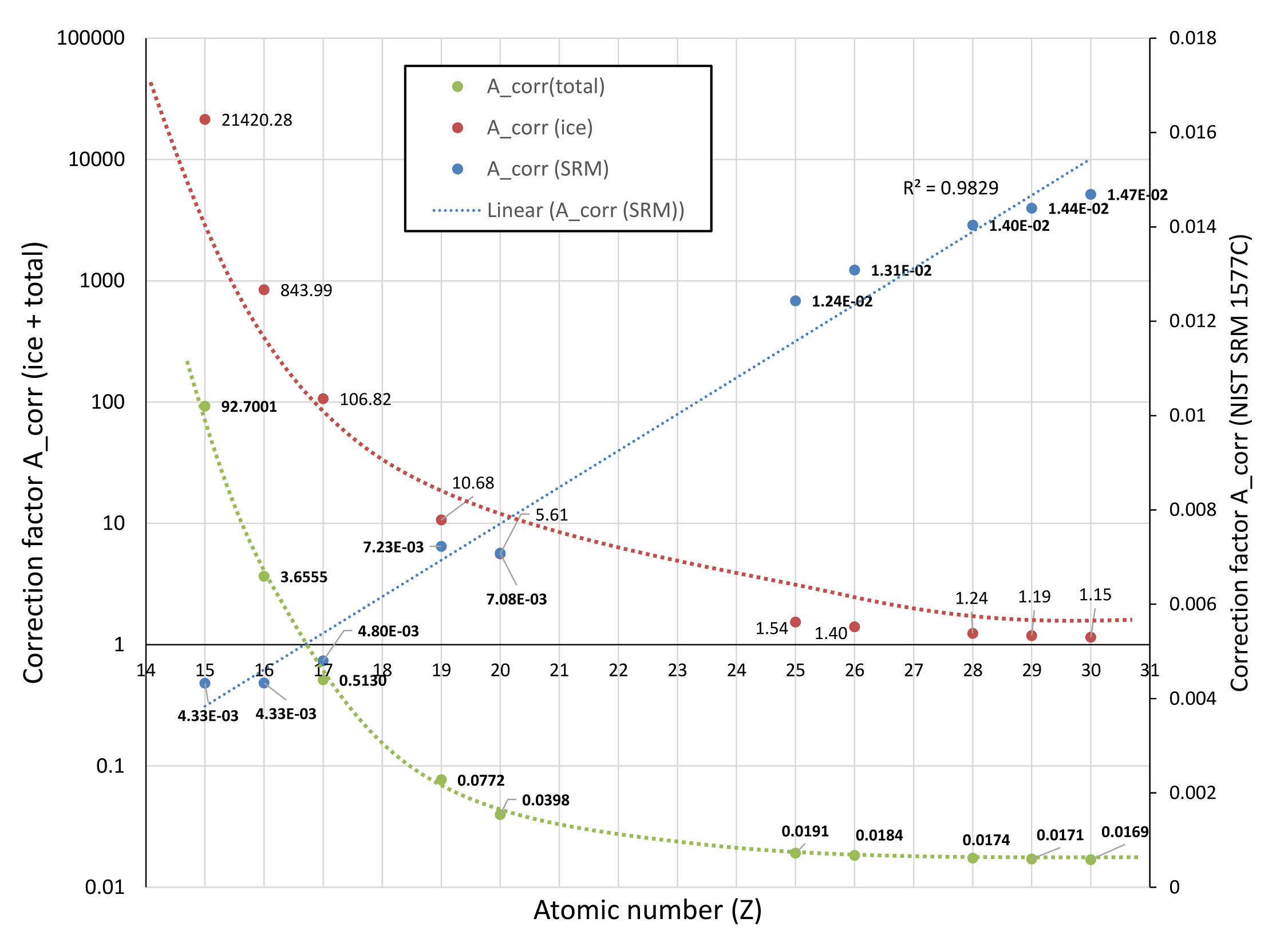

Supplement: S7 Fig — Correction factor for self-absorption in NIST SRM 1577C (blue), in ice (blue) and total absorption correction factor (green). (TIF) [file pone.0190495.s007.tif]

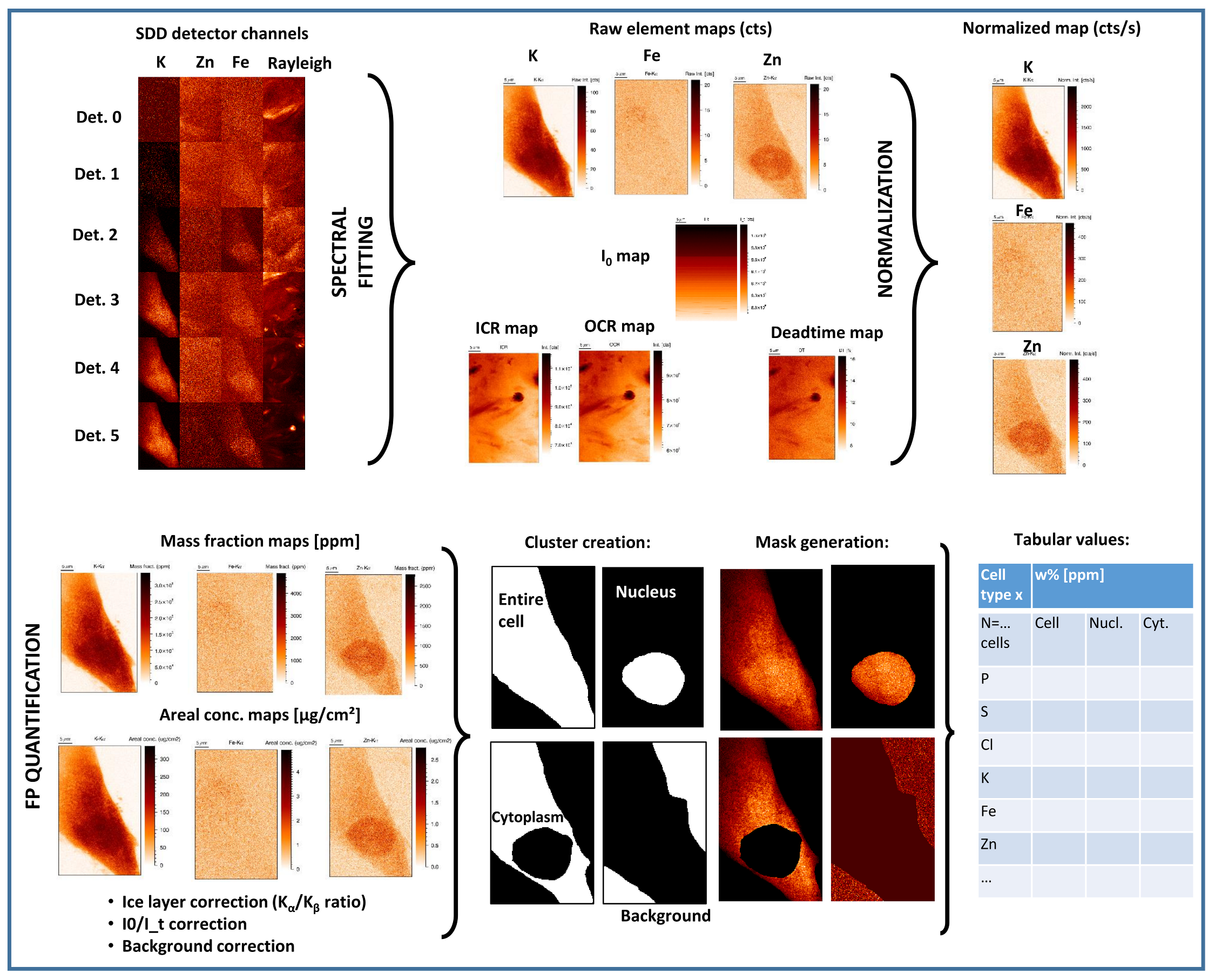

Supplement: S8 Fig — From the incoming (ICR) and outgoing count rate map (OCR), the dead time percentage map is determined (in %). Together with the I0 map (in a.u.) and the raw element intensity maps (in cts), normalized element intensity maps are produced (in cts/s). Using the Fundamental Parameter equation, the normalized element maps are converted into concentration maps (in ppm or in μg/cm2). Absorption effects in NIST SRM 1577C and in the ice layer covering the cells are corrected for. Background is subtracted from the entire concentration map. Cluster maps can then be generated containing regions of interest (ROIs), such as entire cell, cytoplasm and background, omitting hot-spots. In combination with the quantitative element distributions, background corrected mean concentrations of ROIs are obtained. (TIF) [file pone.0190495.s008.tif]
